# Search for a standard-model-like Higgs boson with a mass in the range 145 to 1000 GeV at the LHC

**DOI:** 10.1140/epjc/s10052-013-2469-8

**Published:** 2013-06-14

**Authors:** The CMS Collaboration, S. Chatrchyan, V. Khachatryan, A. M. Sirunyan, A. Tumasyan, W. Adam, T. Bergauer, M. Dragicevic, J. Erö, C. Fabjan, M. Friedl, R. Frühwirth, V. M. Ghete, N. Hörmann, J. Hrubec, M. Jeitler, W. Kiesenhofer, V. Knünz, M. Krammer, I. Krätschmer, D. Liko, I. Mikulec, D. Rabady, B. Rahbaran, C. Rohringer, H. Rohringer, R. Schöfbeck, J. Strauss, A. Taurok, W. Treberer-Treberspurg, W. Waltenberger, C.-E. Wulz, V. Mossolov, N. Shumeiko, J. Suarez Gonzalez, S. Alderweireldt, M. Bansal, S. Bansal, T. Cornelis, E. A. De Wolf, X. Janssen, A. Knutsson, S. Luyckx, L. Mucibello, S. Ochesanu, B. Roland, R. Rougny, H. Van Haevermaet, P. Van Mechelen, N. Van Remortel, A. Van Spilbeeck, F. Blekman, S. Blyweert, J. D’Hondt, A. Kalogeropoulos, J. Keaveney, M. Maes, A. Olbrechts, S. Tavernier, W. Van Doninck, P. Van Mulders, G. P. Van Onsem, I. Villella, B. Clerbaux, G. De Lentdecker, A. P. R. Gay, T. Hreus, A. Léonard, P. E. Marage, A. Mohammadi, T. Reis, L. Thomas, C. Vander Velde, P. Vanlaer, J. Wang, V. Adler, K. Beernaert, L. Benucci, A. Cimmino, S. Costantini, S. Dildick, G. Garcia, B. Klein, J. Lellouch, A. Marinov, J. Mccartin, A. A. Ocampo Rios, D. Ryckbosch, M. Sigamani, N. Strobbe, F. Thyssen, M. Tytgat, S. Walsh, E. Yazgan, N. Zaganidis, S. Basegmez, G. Bruno, R. Castello, L. Ceard, C. Delaere, T. du Pree, D. Favart, L. Forthomme, A. Giammanco, J. Hollar, V. Lemaitre, J. Liao, O. Militaru, C. Nuttens, D. Pagano, A. Pin, K. Piotrzkowski, A. Popov, M. Selvaggi, J. M. Vizan Garcia, N. Beliy, T. Caebergs, E. Daubie, G. H. Hammad, G. A. Alves, M. Correa Martins Junior, T. Martins, M. E. Pol, M. H. G. Souza, W. L. Aldá Júnior, W. Carvalho, J. Chinellato, A. Custódio, E. M. Da Costa, D. De Jesus Damiao, C. De Oliveira Martins, S. Fonseca De Souza, H. Malbouisson, M. Malek, D. Matos Figueiredo, L. Mundim, H. Nogima, W. L. Prado Da Silva, A. Santoro, L. Soares Jorge, A. Sznajder, E. J. Tonelli Manganote, A. Vilela Pereira, T. S. Anjos, C. A. Bernardes, F. A. Dias, T. R. Fernandez Perez Tomei, E. M. Gregores, C. Lagana, F. Marinho, P. G. Mercadante, S. F. Novaes, Sandra S. Padula, V. Genchev, P. Iaydjiev, S. Piperov, M. Rodozov, S. Stoykova, G. Sultanov, V. Tcholakov, R. Trayanov, M. Vutova, A. Dimitrov, R. Hadjiiska, V. Kozhuharov, L. Litov, B. Pavlov, P. Petkov, J. G. Bian, G. M. Chen, H. S. Chen, C. H. Jiang, D. Liang, S. Liang, X. Meng, J. Tao, J. Wang, X. Wang, Z. Wang, H. Xiao, M. Xu, C. Asawatangtrakuldee, Y. Ban, Y. Guo, Q. Li, W. Li, S. Liu, Y. Mao, S. J. Qian, D. Wang, L. Zhang, W. Zou, C. Avila, C. A. Carrillo Montoya, J. P. Gomez, B. Gomez Moreno, J. C. Sanabria, N. Godinovic, D. Lelas, R. Plestina, D. Polic, I. Puljak, Z. Antunovic, M. Kovac, V. Brigljevic, S. Duric, K. Kadija, J. Luetic, D. Mekterovic, S. Morovic, L. Tikvica, A. Attikis, G. Mavromanolakis, J. Mousa, C. Nicolaou, F. Ptochos, P. A. Razis, M. Finger, M. Finger, Y. Assran, A. Ellithi Kamel, M. A. Mahmoud, A. Mahrous, A. Radi, M. Kadastik, M. Müntel, M. Murumaa, M. Raidal, L. Rebane, A. Tiko, P. Eerola, G. Fedi, M. Voutilainen, J. Härkönen, V. Karimäki, R. Kinnunen, M. J. Kortelainen, T. Lampén, K. Lassila-Perini, S. Lehti, T. Lindén, P. Luukka, T. Mäenpää, T. Peltola, E. Tuominen, J. Tuominiemi, E. Tuovinen, L. Wendland, A. Korpela, T. Tuuva, M. Besancon, S. Choudhury, F. Couderc, M. Dejardin, D. Denegri, B. Fabbro, J. L. Faure, F. Ferri, S. Ganjour, A. Givernaud, P. Gras, G. Hamel de Monchenault, P. Jarry, E. Locci, J. Malcles, L. Millischer, A. Nayak, J. Rander, A. Rosowsky, M. Titov, S. Baffioni, F. Beaudette, L. Benhabib, L. Bianchini, M. Bluj, P. Busson, C. Charlot, N. Daci, T. Dahms, M. Dalchenko, L. Dobrzynski, A. Florent, R. Granier de Cassagnac, M. Haguenauer, P. Miné, C. Mironov, I. N. Naranjo, M. Nguyen, C. Ochando, P. Paganini, D. Sabes, R. Salerno, Y. Sirois, C. Veelken, A. Zabi, J.-L. Agram, J. Andrea, D. Bloch, D. Bodin, J.-M. Brom, E. C. Chabert, C. Collard, E. Conte, F. Drouhin, J.-C. Fontaine, D. Gelé, U. Goerlach, C. Goetzmann, P. Juillot, A.-C. Le Bihan, P. Van Hove, S. Beauceron, N. Beaupere, O. Bondu, G. Boudoul, S. Brochet, J. Chasserat, R. Chierici, D. Contardo, P. Depasse, H. El Mamouni, J. Fay, S. Gascon, M. Gouzevitch, B. Ille, T. Kurca, M. Lethuillier, L. Mirabito, S. Perries, L. Sgandurra, V. Sordini, Y. Tschudi, M. Vander Donckt, P. Verdier, S. Viret, Z. Tsamalaidze, C. Autermann, S. Beranek, B. Calpas, M. Edelhoff, L. Feld, N. Heracleous, O. Hindrichs, K. Klein, J. Merz, A. Ostapchuk, A. Perieanu, F. Raupach, J. Sammet, S. Schael, D. Sprenger, H. Weber, B. Wittmer, V. Zhukov, M. Ata, J. Caudron, E. Dietz-Laursonn, D. Duchardt, M. Erdmann, R. Fischer, A. Güth, T. Hebbeker, C. Heidemann, K. Hoepfner, D. Klingebiel, P. Kreuzer, M. Merschmeyer, A. Meyer, M. Olschewski, K. Padeken, P. Papacz, H. Pieta, H. Reithler, S. A. Schmitz, L. Sonnenschein, J. Steggemann, D. Teyssier, S. Thüer, M. Weber, V. Cherepanov, Y. Erdogan, G. Flügge, H. Geenen, M. Geisler, W. Haj Ahmad, F. Hoehle, B. Kargoll, T. Kress, Y. Kuessel, J. Lingemann, A. Nowack, I. M. Nugent, L. Perchalla, O. Pooth, A. Stahl, M. Aldaya Martin, I. Asin, N. Bartosik, J. Behr, W. Behrenhoff, U. Behrens, M. Bergholz, A. Bethani, K. Borras, A. Burgmeier, A. Cakir, L. Calligaris, A. Campbell, F. Costanza, D. Dammann, C. Diez Pardos, T. Dorland, G. Eckerlin, D. Eckstein, G. Flucke, A. Geiser, I. Glushkov, P. Gunnellini, S. Habib, J. Hauk, G. Hellwig, H. Jung, M. Kasemann, P. Katsas, C. Kleinwort, H. Kluge, M. Krämer, D. Krücker, E. Kuznetsova, W. Lange, J. Leonard, K. Lipka, W. Lohmann, B. Lutz, R. Mankel, I. Marfin, M. Marienfeld, I.-A. Melzer-Pellmann, A. B. Meyer, J. Mnich, A. Mussgiller, S. Naumann-Emme, O. Novgorodova, F. Nowak, J. Olzem, H. Perrey, A. Petrukhin, D. Pitzl, A. Raspereza, P. M. Ribeiro Cipriano, C. Riedl, E. Ron, M. Rosin, J. Salfeld-Nebgen, R. Schmidt, T. Schoerner-Sadenius, N. Sen, M. Stein, R. Walsh, C. Wissing, V. Blobel, H. Enderle, J. Erfle, U. Gebbert, M. Görner, M. Gosselink, J. Haller, K. Heine, R. S. Höing, K. Kaschube, G. Kaussen, H. Kirschenmann, R. Klanner, J. Lange, T. Peiffer, N. Pietsch, D. Rathjens, C. Sander, H. Schettler, P. Schleper, E. Schlieckau, A. Schmidt, T. Schum, M. Seidel, J. Sibille, V. Sola, H. Stadie, G. Steinbrück, J. Thomsen, L. Vanelderen, C. Barth, C. Baus, J. Berger, C. Böser, T. Chwalek, W. De Boer, A. Descroix, A. Dierlamm, M. Feindt, M. Guthoff, C. Hackstein, F. Hartmann, T. Hauth, M. Heinrich, H. Held, K. H. Hoffmann, U. Husemann, I. Katkov, J. R. Komaragiri, A. Kornmayer, P. Lobelle Pardo, D. Martschei, S. Mueller, Th. Müller, M. Niegel, A. Nürnberg, O. Oberst, J. Ott, G. Quast, K. Rabbertz, F. Ratnikov, N. Ratnikova, S. Röcker, F.-P. Schilling, G. Schott, H. J. Simonis, F. M. Stober, D. Troendle, R. Ulrich, J. Wagner-Kuhr, S. Wayand, T. Weiler, M. Zeise, G. Anagnostou, G. Daskalakis, T. Geralis, S. Kesisoglou, A. Kyriakis, D. Loukas, A. Markou, C. Markou, E. Ntomari, L. Gouskos, T. J. Mertzimekis, A. Panagiotou, N. Saoulidou, E. Stiliaris, X. Aslanoglou, I. Evangelou, G. Flouris, C. Foudas, P. Kokkas, N. Manthos, I. Papadopoulos, E. Paradas, G. Bencze, C. Hajdu, P. Hidas, D. Horvath, B. Radics, F. Sikler, V. Veszpremi, G. Vesztergombi, A. J. Zsigmond, N. Beni, S. Czellar, J. Molnar, J. Palinkas, Z. Szillasi, J. Karancsi, P. Raics, Z. L. Trocsanyi, B. Ujvari, S. B. Beri, V. Bhatnagar, N. Dhingra, R. Gupta, M. Kaur, M. Z. Mehta, M. Mittal, N. Nishu, L. K. Saini, A. Sharma, J. B. Singh, Ashok Kumar, Arun Kumar, S. Ahuja, A. Bhardwaj, B. C. Choudhary, S. Malhotra, M. Naimuddin, K. Ranjan, P. Saxena, V. Sharma, R. K. Shivpuri, S. Banerjee, S. Bhattacharya, K. Chatterjee, S. Dutta, B. Gomber, Sa. Jain, Sh. Jain, R. Khurana, A. Modak, S. Mukherjee, D. Roy, S. Sarkar, M. Sharan, A. Abdulsalam, D. Dutta, S. Kailas, V. Kumar, A. K. Mohanty, L. M. Pant, P. Shukla, A. Topkar, T. Aziz, R. M. Chatterjee, S. Ganguly, M. Guchait, A. Gurtu, M. Maity, G. Majumder, K. Mazumdar, G. B. Mohanty, B. Parida, K. Sudhakar, N. Wickramage, S. Banerjee, S. Dugad, H. Arfaei, H. Bakhshiansohi, S. M. Etesami, A. Fahim, H. Hesari, A. Jafari, M. Khakzad, M. Mohammadi Najafabadi, S. Paktinat Mehdiabadi, B. Safarzadeh, M. Zeinali, M. Grunewald, M. Abbrescia, L. Barbone, C. Calabria, S. S. Chhibra, A. Colaleo, D. Creanza, N. De Filippis, M. De Palma, L. Fiore, G. Iaselli, G. Maggi, M. Maggi, B. Marangelli, S. My, S. Nuzzo, N. Pacifico, A. Pompili, G. Pugliese, G. Selvaggi, L. Silvestris, G. Singh, R. Venditti, P. Verwilligen, G. Zito, G. Abbiendi, A. C. Benvenuti, D. Bonacorsi, S. Braibant-Giacomelli, L. Brigliadori, R. Campanini, P. Capiluppi, A. Castro, F. R. Cavallo, M. Cuffiani, G. M. Dallavalle, F. Fabbri, A. Fanfani, D. Fasanella, P. Giacomelli, C. Grandi, L. Guiducci, S. Marcellini, G. Masetti, M. Meneghelli, A. Montanari, F. L. Navarria, F. Odorici, A. Perrotta, F. Primavera, A. M. Rossi, T. Rovelli, G. P. Siroli, N. Tosi, R. Travaglini, S. Albergo, M. Chiorboli, S. Costa, R. Potenza, A. Tricomi, C. Tuve, G. Barbagli, V. Ciulli, C. Civinini, R. D’Alessandro, E. Focardi, S. Frosali, E. Gallo, S. Gonzi, P. Lenzi, M. Meschini, S. Paoletti, G. Sguazzoni, A. Tropiano, L. Benussi, S. Bianco, F. Fabbri, D. Piccolo, P. Fabbricatore, R. Musenich, S. Tosi, A. Benaglia, F. De Guio, L. Di Matteo, S. Fiorendi, S. Gennai, A. Ghezzi, P. Govoni, M. T. Lucchini, S. Malvezzi, R. A. Manzoni, A. Martelli, A. Massironi, D. Menasce, L. Moroni, M. Paganoni, D. Pedrini, S. Ragazzi, N. Redaelli, T. Tabarelli de Fatis, S. Buontempo, N. Cavallo, A. De Cosa, O. Dogangun, F. Fabozzi, A. O. M. Iorio, L. Lista, S. Meola, M. Merola, P. Paolucci, P. Azzi, N. Bacchetta, M. Bellato, D. Bisello, A. Branca, R. Carlin, P. Checchia, T. Dorigo, U. Dosselli, S. Fantinel, M. Galanti, F. Gasparini, U. Gasparini, P. Giubilato, A. Gozzelino, K. Kanishchev, S. Lacaprara, I. Lazzizzera, M. Margoni, A. T. Meneguzzo, M. Nespolo, J. Pazzini, N. Pozzobon, P. Ronchese, F. Simonetto, E. Torassa, M. Tosi, S. Vanini, P. Zotto, G. Zumerle, M. Gabusi, S. P. Ratti, C. Riccardi, P. Vitulo, M. Biasini, G. M. Bilei, L. Fanò, P. Lariccia, G. Mantovani, M. Menichelli, A. Nappi, F. Romeo, A. Saha, A. Santocchia, A. Spiezia, P. Azzurri, G. Bagliesi, T. Boccali, G. Broccolo, R. Castaldi, R. T. D’Agnolo, R. Dell’Orso, F. Fiori, L. Foà, A. Giassi, A. Kraan, F. Ligabue, T. Lomtadze, L. Martini, A. Messineo, F. Palla, A. Rizzi, A. T. Serban, P. Spagnolo, P. Squillacioti, R. Tenchini, G. Tonelli, A. Venturi, P. G. Verdini, C. Vernieri, L. Barone, F. Cavallari, D. Del Re, M. Diemoz, C. Fanelli, M. Grassi, E. Longo, F. Margaroli, P. Meridiani, F. Micheli, S. Nourbakhsh, G. Organtini, R. Paramatti, S. Rahatlou, L. Soffi, N. Amapane, R. Arcidiacono, S. Argiro, M. Arneodo, C. Biino, N. Cartiglia, S. Casasso, M. Costa, P. De Remigis, N. Demaria, C. Mariotti, S. Maselli, E. Migliore, V. Monaco, M. Musich, M. M. Obertino, N. Pastrone, M. Pelliccioni, A. Potenza, A. Romero, M. Ruspa, R. Sacchi, A. Solano, A. Staiano, U. Tamponi, S. Belforte, V. Candelise, M. Casarsa, F. Cossutti, G. Della Ricca, B. Gobbo, C. La Licata, M. Marone, D. Montanino, A. Penzo, A. Schizzi, A. Zanetti, T. Y. Kim, S. K. Nam, S. Chang, D. H. Kim, G. N. Kim, J. E. Kim, D. J. Kong, Y. D. Oh, H. Park, D. C. Son, J. Y. Kim, Zero J. Kim, S. Song, S. Choi, D. Gyun, B. Hong, M. Jo, H. Kim, T. J. Kim, K. S. Lee, D. H. Moon, S. K. Park, Y. Roh, M. Choi, J. H. Kim, C. Park, I. C. Park, S. Park, G. Ryu, Y. Choi, Y. K. Choi, J. Goh, M. S. Kim, E. Kwon, B. Lee, J. Lee, S. Lee, H. Seo, I. Yu, I. Grigelionis, A. Juodagalvis, H. Castilla-Valdez, E. De La Cruz-Burelo, I. Heredia-de La Cruz, R. Lopez-Fernandez, J. Martínez-Ortega, A. Sanchez-Hernandez, L. M. Villasenor-Cendejas, S. Carrillo Moreno, F. Vazquez Valencia, H. A. Salazar Ibarguen, E. Casimiro Linares, A. Morelos Pineda, M. A. Reyes-Santos, D. Krofcheck, A. J. Bell, P. H. Butler, R. Doesburg, S. Reucroft, H. Silverwood, M. Ahmad, M. I. Asghar, J. Butt, H. R. Hoorani, S. Khalid, W. A. Khan, T. Khurshid, S. Qazi, M. A. Shah, M. Shoaib, H. Bialkowska, B. Boimska, T. Frueboes, M. Górski, M. Kazana, K. Nawrocki, K. Romanowska-Rybinska, M. Szleper, G. Wrochna, P. Zalewski, G. Brona, K. Bunkowski, M. Cwiok, W. Dominik, K. Doroba, A. Kalinowski, M. Konecki, J. Krolikowski, M. Misiura, W. Wolszczak, N. Almeida, P. Bargassa, A. David, P. Faccioli, P. G. Ferreira Parracho, M. Gallinaro, J. Seixas, J. Varela, P. Vischia, P. Bunin, I. Golutvin, I. Gorbunov, V. Karjavin, V. Konoplyanikov, G. Kozlov, A. Lanev, A. Malakhov, P. Moisenz, V. Palichik, V. Perelygin, M. Savina, S. Shmatov, S. Shulha, V. Smirnov, A. Volodko, A. Zarubin, S. Evstyukhin, V. Golovtsov, Y. Ivanov, V. Kim, P. Levchenko, V. Murzin, V. Oreshkin, I. Smirnov, V. Sulimov, L. Uvarov, S. Vavilov, A. Vorobyev, An. Vorobyev, Yu. Andreev, A. Dermenev, S. Gninenko, N. Golubev, M. Kirsanov, N. Krasnikov, V. Matveev, A. Pashenkov, D. Tlisov, A. Toropin, V. Epshteyn, M. Erofeeva, V. Gavrilov, N. Lychkovskaya, V. Popov, G. Safronov, S. Semenov, A. Spiridonov, V. Stolin, E. Vlasov, A. Zhokin, V. Andreev, M. Azarkin, I. Dremin, M. Kirakosyan, A. Leonidov, G. Mesyats, S. V. Rusakov, A. Vinogradov, A. Belyaev, E. Boos, V. Bunichev, M. Dubinin, L. Dudko, A. Gribushin, V. Klyukhin, O. Kodolova, I. Lokhtin, A. Markina, S. Obraztsov, S. Petrushanko, V. Savrin, A. Snigirev, I. Azhgirey, I. Bayshev, S. Bitioukov, V. Kachanov, A. Kalinin, D. Konstantinov, V. Krychkine, V. Petrov, R. Ryutin, A. Sobol, L. Tourtchanovitch, S. Troshin, N. Tyurin, A. Uzunian, A. Volkov, P. Adzic, M. Ekmedzic, D. Krpic, J. Milosevic, M. Aguilar-Benitez, J. Alcaraz Maestre, C. Battilana, E. Calvo, M. Cerrada, M. Chamizo Llatas, N. Colino, B. De La Cruz, A. Delgado Peris, D. Domínguez Vázquez, C. Fernandez Bedoya, J. P. Fernández Ramos, A. Ferrando, J. Flix, M. C. Fouz, P. Garcia-Abia, O. Gonzalez Lopez, S. Goy Lopez, J. M. Hernandez, M. I. Josa, G. Merino, E. Navarro De Martino, J. Puerta Pelayo, A. Quintario Olmeda, I. Redondo, L. Romero, J. Santaolalla, M. S. Soares, C. Willmott, C. Albajar, J. F. de Trocóniz, H. Brun, J. Cuevas, J. Fernandez Menendez, S. Folgueras, I. Gonzalez Caballero, L. Lloret Iglesias, J. Piedra Gomez, J. A. Brochero Cifuentes, I. J. Cabrillo, A. Calderon, S. H. Chuang, J. Duarte Campderros, M. Fernandez, G. Gomez, J. Gonzalez Sanchez, A. Graziano, C. Jorda, A. Lopez Virto, J. Marco, R. Marco, C. Martinez Rivero, F. Matorras, F. J. Munoz Sanchez, T. Rodrigo, A. Y. Rodríguez-Marrero, A. Ruiz-Jimeno, L. Scodellaro, I. Vila, R. Vilar Cortabitarte, D. Abbaneo, E. Auffray, G. Auzinger, M. Bachtis, P. Baillon, A. H. Ball, D. Barney, J. Bendavid, J. F. Benitez, C. Bernet, G. Bianchi, P. Bloch, A. Bocci, A. Bonato, C. Botta, H. Breuker, T. Camporesi, G. Cerminara, T. Christiansen, J. A. Coarasa Perez, S. Colafranceschi, D. d’Enterria, A. Dabrowski, A. De Roeck, S. De Visscher, S. Di Guida, M. Dobson, N. Dupont-Sagorin, A. Elliott-Peisert, J. Eugster, W. Funk, G. Georgiou, M. Giffels, D. Gigi, K. Gill, D. Giordano, M. Girone, M. Giunta, F. Glege, R. Gomez-Reino Garrido, S. Gowdy, R. Guida, J. Hammer, M. Hansen, P. Harris, C. Hartl, B. Hegner, A. Hinzmann, V. Innocente, P. Janot, K. Kaadze, E. Karavakis, K. Kousouris, K. Krajczar, P. Lecoq, Y.-J. Lee, C. Lourenço, N. Magini, M. Malberti, L. Malgeri, M. Mannelli, L. Masetti, F. Meijers, S. Mersi, E. Meschi, R. Moser, M. Mulders, P. Musella, E. Nesvold, L. Orsini, E. Palencia Cortezon, E. Perez, L. Perrozzi, A. Petrilli, A. Pfeiffer, M. Pierini, M. Pimiä, D. Piparo, G. Polese, L. Quertenmont, A. Racz, W. Reece, J. Rodrigues Antunes, G. Rolandi, C. Rovelli, M. Rovere, H. Sakulin, F. Santanastasio, C. Schäfer, C. Schwick, I. Segoni, S. Sekmen, A. Sharma, P. Siegrist, P. Silva, M. Simon, P. Sphicas, D. Spiga, M. Stoye, A. Tsirou, G. I. Veres, J. R. Vlimant, H. K. Wöhri, S. D. Worm, W. D. Zeuner, W. Bertl, K. Deiters, W. Erdmann, K. Gabathuler, R. Horisberger, Q. Ingram, H. C. Kaestli, S. König, D. Kotlinski, U. Langenegger, F. Meier, D. Renker, T. Rohe, F. Bachmair, L. Bäni, P. Bortignon, M. A. Buchmann, B. Casal, N. Chanon, A. Deisher, G. Dissertori, M. Dittmar, M. Donegà, M. Dünser, P. Eller, C. Grab, D. Hits, P. Lecomte, W. Lustermann, A. C. Marini, P. Martinez Ruiz del Arbol, N. Mohr, F. Moortgat, C. Nägeli, P. Nef, F. Nessi-Tedaldi, F. Pandolfi, L. Pape, F. Pauss, M. Peruzzi, F. J. Ronga, M. Rossini, L. Sala, A. K. Sanchez, A. Starodumov, B. Stieger, M. Takahashi, L. Tauscher, A. Thea, K. Theofilatos, D. Treille, C. Urscheler, R. Wallny, H. A. Weber, C. Amsler, V. Chiochia, C. Favaro, M. Ivova Rikova, B. Kilminster, B. Millan Mejias, P. Otiougova, P. Robmann, H. Snoek, S. Taroni, S. Tupputi, M. Verzetti, M. Cardaci, K. H. Chen, C. Ferro, C. M. Kuo, S. W. Li, W. Lin, Y. J. Lu, R. Volpe, S. S. Yu, P. Bartalini, P. Chang, Y. H. Chang, Y. W. Chang, Y. Chao, K. F. Chen, C. Dietz, U. Grundler, W.-S. Hou, Y. Hsiung, K. Y. Kao, Y. J. Lei, R.-S. Lu, D. Majumder, E. Petrakou, X. Shi, J. G. Shiu, Y. M. Tzeng, M. Wang, B. Asavapibhop, N. Suwonjandee, A. Adiguzel, M. N. Bakirci, S. Cerci, C. Dozen, I. Dumanoglu, E. Eskut, S. Girgis, G. Gokbulut, E. Gurpinar, I. Hos, E. E. Kangal, A. Kayis Topaksu, G. Onengut, K. Ozdemir, S. Ozturk, A. Polatoz, K. Sogut, D. Sunar Cerci, B. Tali, H. Topakli, M. Vergili, I. V. Akin, T. Aliev, B. Bilin, S. Bilmis, M. Deniz, H. Gamsizkan, A. M. Guler, G. Karapinar, K. Ocalan, A. Ozpineci, M. Serin, R. Sever, U. E. Surat, M. Yalvac, M. Zeyrek, E. Gülmez, B. Isildak, M. Kaya, O. Kaya, S. Ozkorucuklu, N. Sonmez, H. Bahtiyar, E. Barlas, K. Cankocak, Y. O. Günaydin, F. I. Vardarlı, M. Yücel, L. Levchuk, P. Sorokin, J. J. Brooke, E. Clement, D. Cussans, H. Flacher, R. Frazier, J. Goldstein, M. Grimes, G. P. Heath, H. F. Heath, L. Kreczko, S. Metson, D. M. Newbold, K. Nirunpong, A. Poll, S. Senkin, V. J. Smith, T. Williams, L. Basso, K. W. Bell, A. Belyaev, C. Brew, R. M. Brown, D. J. A. Cockerill, J. A. Coughlan, K. Harder, S. Harper, J. Jackson, E. Olaiya, D. Petyt, B. C. Radburn-Smith, C. H. Shepherd-Themistocleous, I. R. Tomalin, W. J. Womersley, R. Bainbridge, G. Ball, O. Buchmuller, D. Burton, D. Colling, N. Cripps, M. Cutajar, P. Dauncey, G. Davies, M. Della Negra, W. Ferguson, J. Fulcher, D. Futyan, A. Gilbert, A. Guneratne Bryer, G. Hall, Z. Hatherell, J. Hays, G. Iles, M. Jarvis, G. Karapostoli, M. Kenzie, R. Lane, R. Lucas, L. Lyons, A.-M. Magnan, J. Marrouche, B. Mathias, R. Nandi, J. Nash, A. Nikitenko, J. Pela, M. Pesaresi, K. Petridis, M. Pioppi, D. M. Raymond, S. Rogerson, A. Rose, C. Seez, P. Sharp, A. Sparrow, A. Tapper, M. Vazquez Acosta, T. Virdee, S. Wakefield, N. Wardle, T. Whyntie, M. Chadwick, J. E. Cole, P. R. Hobson, A. Khan, P. Kyberd, D. Leggat, D. Leslie, W. Martin, I. D. Reid, P. Symonds, L. Teodorescu, M. Turner, J. Dittmann, K. Hatakeyama, A. Kasmi, H. Liu, T. Scarborough, O. Charaf, S. I. Cooper, C. Henderson, P. Rumerio, A. Avetisyan, T. Bose, C. Fantasia, A. Heister, P. Lawson, D. Lazic, J. Rohlf, D. Sperka, J. St. John, L. Sulak, J. Alimena, S. Bhattacharya, G. Christopher, D. Cutts, Z. Demiragli, A. Ferapontov, A. Garabedian, U. Heintz, G. Kukartsev, E. Laird, G. Landsberg, M. Luk, M. Narain, M. Segala, T. Sinthuprasith, T. Speer, R. Breedon, G. Breto, M. Calderon De La Barca Sanchez, S. Chauhan, M. Chertok, J. Conway, R. Conway, P. T. Cox, R. Erbacher, M. Gardner, R. Houtz, W. Ko, A. Kopecky, R. Lander, O. Mall, T. Miceli, R. Nelson, D. Pellett, F. Ricci-Tam, B. Rutherford, M. Searle, J. Smith, M. Squires, M. Tripathi, R. Yohay, V. Andreev, D. Cline, R. Cousins, S. Erhan, P. Everaerts, C. Farrell, M. Felcini, J. Hauser, M. Ignatenko, C. Jarvis, G. Rakness, P. Schlein, P. Traczyk, V. Valuev, M. Weber, J. Babb, R. Clare, M. E. Dinardo, J. Ellison, J. W. Gary, F. Giordano, G. Hanson, H. Liu, O. R. Long, A. Luthra, H. Nguyen, S. Paramesvaran, J. Sturdy, S. Sumowidagdo, R. Wilken, S. Wimpenny, W. Andrews, J. G. Branson, G. B. Cerati, S. Cittolin, D. Evans, A. Holzner, R. Kelley, M. Lebourgeois, J. Letts, I. Macneill, B. Mangano, S. Padhi, C. Palmer, G. Petrucciani, M. Pieri, M. Sani, V. Sharma, S. Simon, E. Sudano, M. Tadel, Y. Tu, A. Vartak, S. Wasserbaech, F. Würthwein, A. Yagil, J. Yoo, D. Barge, R. Bellan, C. Campagnari, M. D’Alfonso, T. Danielson, K. Flowers, P. Geffert, C. George, F. Golf, J. Incandela, C. Justus, P. Kalavase, D. Kovalskyi, V. Krutelyov, S. Lowette, R. Magaña Villalba, N. Mccoll, V. Pavlunin, J. Ribnik, J. Richman, R. Rossin, D. Stuart, W. To, C. West, A. Apresyan, A. Bornheim, J. Bunn, Y. Chen, E. Di Marco, J. Duarte, D. Kcira, Y. Ma, A. Mott, H. B. Newman, C. Rogan, M. Spiropulu, V. Timciuc, J. Veverka, R. Wilkinson, S. Xie, Y. Yang, R. Y. Zhu, V. Azzolini, A. Calamba, R. Carroll, T. Ferguson, Y. Iiyama, D. W. Jang, Y. F. Liu, M. Paulini, J. Russ, H. Vogel, I. Vorobiev, J. P. Cumalat, B. R. Drell, W. T. Ford, A. Gaz, E. Luiggi Lopez, U. Nauenberg, J. G. Smith, K. Stenson, K. A. Ulmer, S. R. Wagner, J. Alexander, A. Chatterjee, N. Eggert, L. K. Gibbons, W. Hopkins, A. Khukhunaishvili, B. Kreis, N. Mirman, G. Nicolas Kaufman, J. R. Patterson, A. Ryd, E. Salvati, W. Sun, W. D. Teo, J. Thom, J. Thompson, J. Tucker, Y. Weng, L. Winstrom, P. Wittich, D. Winn, S. Abdullin, M. Albrow, J. Anderson, G. Apollinari, L. A. T. Bauerdick, A. Beretvas, J. Berryhill, P. C. Bhat, K. Burkett, J. N. Butler, V. Chetluru, H. W. K. Cheung, F. Chlebana, S. Cihangir, V. D. Elvira, I. Fisk, J. Freeman, Y. Gao, E. Gottschalk, L. Gray, D. Green, O. Gutsche, R. M. Harris, J. Hirschauer, B. Hooberman, S. Jindariani, M. Johnson, U. Joshi, B. Klima, S. Kunori, S. Kwan, J. Linacre, D. Lincoln, R. Lipton, J. Lykken, K. Maeshima, J. M. Marraffino, V. I. Martinez Outschoorn, S. Maruyama, D. Mason, P. McBride, K. Mishra, S. Mrenna, Y. Musienko, C. Newman-Holmes, V. O’Dell, O. Prokofyev, E. Sexton-Kennedy, S. Sharma, W. J. Spalding, L. Spiegel, L. Taylor, S. Tkaczyk, N. V. Tran, L. Uplegger, E. W. Vaandering, R. Vidal, J. Whitmore, W. Wu, F. Yang, J. C. Yun, D. Acosta, P. Avery, D. Bourilkov, M. Chen, T. Cheng, S. Das, M. De Gruttola, G. P. Di Giovanni, D. Dobur, A. Drozdetskiy, R. D. Field, M. Fisher, Y. Fu, I. K. Furic, J. Hugon, B. Kim, J. Konigsberg, A. Korytov, A. Kropivnitskaya, T. Kypreos, J. F. Low, K. Matchev, P. Milenovic, G. Mitselmakher, L. Muniz, R. Remington, A. Rinkevicius, N. Skhirtladze, M. Snowball, J. Yelton, M. Zakaria, V. Gaultney, S. Hewamanage, L. M. Lebolo, S. Linn, P. Markowitz, G. Martinez, J. L. Rodriguez, T. Adams, A. Askew, J. Bochenek, J. Chen, B. Diamond, S. V. Gleyzer, J. Haas, S. Hagopian, V. Hagopian, K. F. Johnson, H. Prosper, V. Veeraraghavan, M. Weinberg, M. M. Baarmand, B. Dorney, M. Hohlmann, H. Kalakhety, F. Yumiceva, M. R. Adams, L. Apanasevich, V. E. Bazterra, R. R. Betts, I. Bucinskaite, J. Callner, R. Cavanaugh, O. Evdokimov, L. Gauthier, C. E. Gerber, D. J. Hofman, S. Khalatyan, P. Kurt, F. Lacroix, C. O’Brien, C. Silkworth, D. Strom, P. Turner, N. Varelas, U. Akgun, E. A. Albayrak, B. Bilki, W. Clarida, K. Dilsiz, F. Duru, S. Griffiths, J.-P. Merlo, H. Mermerkaya, A. Mestvirishvili, A. Moeller, J. Nachtman, C. R. Newsom, H. Ogul, Y. Onel, F. Ozok, S. Sen, P. Tan, E. Tiras, J. Wetzel, T. Yetkin, K. Yi, B. A. Barnett, B. Blumenfeld, S. Bolognesi, D. Fehling, G. Giurgiu, A. V. Gritsan, G. Hu, P. Maksimovic, M. Swartz, A. Whitbeck, P. Baringer, A. Bean, G. Benelli, R. P. Kenny III, M. Murray, D. Noonan, S. Sanders, R. Stringer, J. S. Wood, A. F. Barfuss, I. Chakaberia, A. Ivanov, S. Khalil, M. Makouski, Y. Maravin, S. Shrestha, I. Svintradze, J. Gronberg, D. Lange, F. Rebassoo, D. Wright, A. Baden, B. Calvert, S. C. Eno, J. A. Gomez, N. J. Hadley, R. G. Kellogg, T. Kolberg, Y. Lu, M. Marionneau, A. C. Mignerey, K. Pedro, A. Peterman, A. Skuja, J. Temple, M. B. Tonjes, S. C. Tonwar, A. Apyan, G. Bauer, W. Busza, E. Butz, I. A. Cali, M. Chan, V. Dutta, G. Gomez Ceballos, M. Goncharov, Y. Kim, M. Klute, A. Levin, P. D. Luckey, T. Ma, S. Nahn, C. Paus, D. Ralph, C. Roland, G. Roland, G. S. F. Stephans, F. Stöckli, K. Sumorok, K. Sung, D. Velicanu, R. Wolf, B. Wyslouch, M. Yang, Y. Yilmaz, A. S. Yoon, M. Zanetti, V. Zhukova, B. Dahmes, A. De Benedetti, G. Franzoni, A. Gude, J. Haupt, S. C. Kao, K. Klapoetke, Y. Kubota, J. Mans, N. Pastika, R. Rusack, M. Sasseville, A. Singovsky, N. Tambe, J. Turkewitz, L. M. Cremaldi, R. Kroeger, L. Perera, R. Rahmat, D. A. Sanders, D. Summers, E. Avdeeva, K. Bloom, S. Bose, D. R. Claes, A. Dominguez, M. Eads, R. Gonzalez Suarez, J. Keller, I. Kravchenko, J. Lazo-Flores, S. Malik, G. R. Snow, J. Dolen, A. Godshalk, I. Iashvili, S. Jain, A. Kharchilava, A. Kumar, S. Rappoccio, Z. Wan, G. Alverson, E. Barberis, D. Baumgartel, M. Chasco, J. Haley, D. Nash, T. Orimoto, D. Trocino, D. Wood, J. Zhang, A. Anastassov, K. A. Hahn, A. Kubik, L. Lusito, N. Mucia, N. Odell, B. Pollack, A. Pozdnyakov, M. Schmitt, S. Stoynev, M. Velasco, S. Won, D. Berry, A. Brinkerhoff, K. M. Chan, M. Hildreth, C. Jessop, D. J. Karmgard, J. Kolb, K. Lannon, W. Luo, S. Lynch, N. Marinelli, D. M. Morse, T. Pearson, M. Planer, R. Ruchti, J. Slaunwhite, N. Valls, M. Wayne, M. Wolf, L. Antonelli, B. Bylsma, L. S. Durkin, C. Hill, R. Hughes, K. Kotov, T. Y. Ling, D. Puigh, M. Rodenburg, G. Smith, C. Vuosalo, G. Williams, B. L. Winer, H. Wolfe, E. Berry, P. Elmer, V. Halyo, P. Hebda, J. Hegeman, A. Hunt, P. Jindal, S. A. Koay, D. Lopes Pegna, P. Lujan, D. Marlow, T. Medvedeva, M. Mooney, J. Olsen, P. Piroué, X. Quan, A. Raval, H. Saka, D. Stickland, C. Tully, J. S. Werner, S. C. Zenz, A. Zuranski, E. Brownson, A. Lopez, H. Mendez, J. E. Ramirez Vargas, E. Alagoz, D. Benedetti, G. Bolla, D. Bortoletto, M. De Mattia, A. Everett, Z. Hu, M. Jones, O. Koybasi, M. Kress, N. Leonardo, V. Maroussov, P. Merkel, D. H. Miller, N. Neumeister, I. Shipsey, D. Silvers, A. Svyatkovskiy, M. Vidal Marono, H. D. Yoo, J. Zablocki, Y. Zheng, S. Guragain, N. Parashar, A. Adair, B. Akgun, K. M. Ecklund, F. J. M. Geurts, W. Li, B. P. Padley, R. Redjimi, J. Roberts, J. Zabel, B. Betchart, A. Bodek, R. Covarelli, P. de Barbaro, R. Demina, Y. Eshaq, T. Ferbel, A. Garcia-Bellido, P. Goldenzweig, J. Han, A. Harel, D. C. Miner, G. Petrillo, D. Vishnevskiy, M. Zielinski, A. Bhatti, R. Ciesielski, L. Demortier, K. Goulianos, G. Lungu, S. Malik, C. Mesropian, S. Arora, A. Barker, J. P. Chou, C. Contreras-Campana, E. Contreras-Campana, D. Duggan, D. Ferencek, Y. Gershtein, R. Gray, E. Halkiadakis, D. Hidas, A. Lath, S. Panwalkar, M. Park, R. Patel, V. Rekovic, J. Robles, K. Rose, S. Salur, S. Schnetzer, C. Seitz, S. Somalwar, R. Stone, M. Walker, G. Cerizza, M. Hollingsworth, S. Spanier, Z. C. Yang, A. York, R. Eusebi, W. Flanagan, J. Gilmore, T. Kamon, V. Khotilovich, R. Montalvo, I. Osipenkov, Y. Pakhotin, A. Perloff, J. Roe, A. Safonov, T. Sakuma, I. Suarez, A. Tatarinov, D. Toback, N. Akchurin, J. Damgov, C. Dragoiu, P. R. Dudero, C. Jeong, K. Kovitanggoon, S. W. Lee, T. Libeiro, I. Volobouev, E. Appelt, A. G. Delannoy, S. Greene, A. Gurrola, W. Johns, C. Maguire, Y. Mao, A. Melo, M. Sharma, P. Sheldon, B. Snook, S. Tuo, J. Velkovska, M. W. Arenton, M. Balazs, S. Boutle, B. Cox, B. Francis, J. Goodell, R. Hirosky, A. Ledovskoy, C. Lin, C. Neu, J. Wood, S. Gollapinni, R. Harr, P. E. Karchin, C. Kottachchi Kankanamge Don, P. Lamichhane, A. Sakharov, M. Anderson, D. A. Belknap, L. Borrello, D. Carlsmith, M. Cepeda, S. Dasu, E. Friis, K. S. Grogg, M. Grothe, R. Hall-Wilton, M. Herndon, A. Hervé, P. Klabbers, J. Klukas, A. Lanaro, C. Lazaridis, R. Loveless, A. Mohapatra, M. U. Mozer, I. Ojalvo, G. A. Pierro, I. Ross, A. Savin, W. H. Smith, J. Swanson

**Affiliations:** 1 CERN, Geneva, Switzerland; 2 Yerevan Physics Institute, Yerevan, Armenia; 3 Institut für Hochenergiephysik der OeAW, Wien, Austria; 4 National Centre for Particle and High Energy Physics, Minsk, Belarus; 5 Universiteit Antwerpen, Antwerpen, Belgium; 6 Vrije Universiteit Brussel, Brussel, Belgium; 7 Université Libre de Bruxelles, Bruxelles, Belgium; 8 Ghent University, Ghent, Belgium; 9 Université Catholique de Louvain, Louvain-la-Neuve, Belgium; 10 Université de Mons, Mons, Belgium; 11 Centro Brasileiro de Pesquisas Fisicas, Rio de Janeiro, Brazil; 12 Universidade do Estado do Rio de Janeiro, Rio de Janeiro, Brazil; 13 Universidade Estadual Paulista, São Paulo, Brazil; 14 Universidade Federal do ABC, São Paulo, Brazil; 15 Institute for Nuclear Research and Nuclear Energy, Sofia, Bulgaria; 16 University of Sofia, Sofia, Bulgaria; 17 Institute of High Energy Physics, Beijing, China; 18 State Key Laboratory of Nuclear Physics and Technology, Peking University, Beijing, China; 19 Universidad de Los Andes, Bogota, Colombia; 20 Technical University of Split, Split, Croatia; 21 University of Split, Split, Croatia; 22 Institute Rudjer Boskovic, Zagreb, Croatia; 23 University of Cyprus, Nicosia, Cyprus; 24 Charles University, Prague, Czech Republic; 25 Academy of Scientific Research and Technology of the Arab Republic of Egypt, Egyptian Network of High Energy Physics, Cairo, Egypt; 26 National Institute of Chemical Physics and Biophysics, Tallinn, Estonia; 27 Department of Physics, University of Helsinki, Helsinki, Finland; 28 Helsinki Institute of Physics, Helsinki, Finland; 29 Lappeenranta University of Technology, Lappeenranta, Finland; 30 DSM/IRFU, CEA/Saclay, Gif-sur-Yvette, France; 31 Laboratoire Leprince-Ringuet, Ecole Polytechnique, IN2P3-CNRS, Palaiseau, France; 32 Institut Pluridisciplinaire Hubert Curien, Université de Strasbourg, Université de Haute Alsace Mulhouse, CNRS/IN2P3, Strasbourg, France; 33 CNRS-IN2P3, Institut de Physique Nucléaire de Lyon, Université de Lyon, Université Claude Bernard Lyon 1, Villeurbanne, France; 34 Institute of High Energy Physics and Informatization, Tbilisi State University, Tbilisi, Georgia; 35 I. Physikalisches Institut, RWTH Aachen University, Aachen, Germany; 36 III. Physikalisches Institut A, RWTH Aachen University, Aachen, Germany; 37 III. Physikalisches Institut B, RWTH Aachen University, Aachen, Germany; 38 Deutsches Elektronen-Synchrotron, Hamburg, Germany; 39 University of Hamburg, Hamburg, Germany; 40 Institut für Experimentelle Kernphysik, Karlsruhe, Germany; 41 Institute of Nuclear and Particle Physics (INPP), NCSR Demokritos, Aghia Paraskevi, Greece; 42 University of Athens, Athens, Greece; 43 University of Ioánnina, Ioánnina, Greece; 44 KFKI Research Institute for Particle and Nuclear Physics, Budapest, Hungary; 45 Institute of Nuclear Research ATOMKI, Debrecen, Hungary; 46 University of Debrecen, Debrecen, Hungary; 47 Panjab University, Chandigarh, India; 48 University of Delhi, Delhi, India; 49 Saha Institute of Nuclear Physics, Kolkata, India; 50 Bhabha Atomic Research Centre, Mumbai, India; 51 Tata Institute of Fundamental Research - EHEP, Mumbai, India; 52 Tata Institute of Fundamental Research - HECR, Mumbai, India; 53 Institute for Research in Fundamental Sciences (IPM), Tehran, Iran; 54 University College Dublin, Dublin, Ireland; 55 INFN Sezione di Bari, Bari, Italy; 56 Università di Bari, Bari, Italy; 57 Politecnico di Bari, Bari, Italy; 58 INFN Sezione di Bologna, Bologna, Italy; 59 Università di Bologna, Bologna, Italy; 60 INFN Sezione di Catania, Catania, Italy; 61 Università di Catania, Catania, Italy; 62 INFN Sezione di Firenze, Firenze, Italy; 63 Università di Firenze, Firenze, Italy; 64 INFN Laboratori Nazionali di Frascati, Frascati, Italy; 65 INFN Sezione di Genova, Genova, Italy; 66 Università di Genova, Genova, Italy; 67 INFN Sezione di Milano-Bicocca, Milano, Italy; 68 Università di Milano-Bicocca, Milano, Italy; 69 INFN Sezione di Napoli, Napoli, Italy; 70 Università di Napoli ’Federico II’, Napoli, Italy; 71 Università della Basilicata (Potenza), Napoli, Italy; 72 Università G. Marconi (Roma), Napoli, Italy; 73 INFN Sezione di Padova, Padova, Italy; 74 Università di Padova, Padova, Italy; 75 Università di Trento (Trento), Padova, Italy; 76 INFN Sezione di Pavia, Pavia, Italy; 77 Università di Pavia, Pavia, Italy; 78 INFN Sezione di Perugia, Perugia, Italy; 79 Università di Perugia, Perugia, Italy; 80 INFN Sezione di Pisa, Pisa, Italy; 81 Università di Pisa, Pisa, Italy; 82 Scuola Normale Superiore di Pisa, Pisa, Italy; 83 INFN Sezione di Roma, Roma, Italy; 84 Università di Roma, Roma, Italy; 85 INFN Sezione di Torino, Torino, Italy; 86 Università di Torino, Torino, Italy; 87 Università del Piemonte Orientale (Novara), Torino, Italy; 88 INFN Sezione di Trieste, Trieste, Italy; 89 Università di Trieste, Trieste, Italy; 90 Kangwon National University, Chunchon, Korea; 91 Kyungpook National University, Daegu, Korea; 92 Institute for Universe and Elementary Particles, Chonnam National University, Kwangju, Korea; 93 Korea University, Seoul, Korea; 94 University of Seoul, Seoul, Korea; 95 Sungkyunkwan University, Suwon, Korea; 96 Vilnius University, Vilnius, Lithuania; 97 Centro de Investigacion y de Estudios Avanzados del IPN, Mexico City, Mexico; 98 Universidad Iberoamericana, Mexico City, Mexico; 99 Benemerita Universidad Autonoma de Puebla, Puebla, Mexico; 100 Universidad Autónoma de San Luis Potosí, San Luis Potosí, Mexico; 101 University of Auckland, Auckland, New Zealand; 102 University of Canterbury, Christchurch, New Zealand; 103 National Centre for Physics, Quaid-I-Azam University, Islamabad, Pakistan; 104 National Centre for Nuclear Research, Swierk, Poland; 105 Institute of Experimental Physics, Faculty of Physics, University of Warsaw, Warsaw, Poland; 106 Laboratório de Instrumentação e Física Experimental de Partículas, Lisboa, Portugal; 107 Joint Institute for Nuclear Research, Dubna, Russia; 108 Petersburg Nuclear Physics Institute, Gatchina (St. Petersburg), Russia; 109 Institute for Nuclear Research, Moscow, Russia; 110 Institute for Theoretical and Experimental Physics, Moscow, Russia; 111 P.N. Lebedev Physical Institute, Moscow, Russia; 112 Skobeltsyn Institute of Nuclear Physics, Lomonosov Moscow State University, Moscow, Russia; 113 State Research Center of Russian Federation, Institute for High Energy Physics, Protvino, Russia; 114 Faculty of Physics and Vinca Institute of Nuclear Sciences, University of Belgrade, Belgrade, Serbia; 115 Centro de Investigaciones Energéticas Medioambientales y Tecnológicas (CIEMAT), Madrid, Spain; 116 Universidad Autónoma de Madrid, Madrid, Spain; 117 Universidad de Oviedo, Oviedo, Spain; 118 Instituto de Física de Cantabria (IFCA), CSIC-Universidad de Cantabria, Santander, Spain; 119 European Organization for Nuclear Research, CERN, Geneva, Switzerland; 120 Paul Scherrer Institut, Villigen, Switzerland; 121 Institute for Particle Physics, ETH Zurich, Zurich, Switzerland; 122 Universität Zürich, Zurich, Switzerland; 123 National Central University, Chung-Li, Taiwan; 124 National Taiwan University (NTU), Taipei, Taiwan; 125 Chulalongkorn University, Bangkok, Thailand; 126 Cukurova University, Adana, Turkey; 127 Physics Department, Middle East Technical University, Ankara, Turkey; 128 Bogazici University, Istanbul, Turkey; 129 Istanbul Technical University, Istanbul, Turkey; 130 National Scientific Center, Kharkov Institute of Physics and Technology, Kharkov, Ukraine; 131 University of Bristol, Bristol, United Kingdom; 132 Rutherford Appleton Laboratory, Didcot, United Kingdom; 133 Imperial College, London, United Kingdom; 134 Brunel University, Uxbridge, United Kingdom; 135 Baylor University, Waco, USA; 136 The University of Alabama, Tuscaloosa, USA; 137 Boston University, Boston, USA; 138 Brown University, Providence, USA; 139 University of California, Davis, Davis, USA; 140 University of California, Los Angeles, USA; 141 University of California, Riverside, Riverside, USA; 142 University of California, San Diego, La Jolla, USA; 143 University of California, Santa Barbara, Santa Barbara, USA; 144 California Institute of Technology, Pasadena, USA; 145 Carnegie Mellon University, Pittsburgh, USA; 146 University of Colorado at Boulder, Boulder, USA; 147 Cornell University, Ithaca, USA; 148 Fairfield University, Fairfield, USA; 149 Fermi National Accelerator Laboratory, Batavia, USA; 150 University of Florida, Gainesville, USA; 151 Florida International University, Miami, USA; 152 Florida State University, Tallahassee, USA; 153 Florida Institute of Technology, Melbourne, USA; 154 University of Illinois at Chicago (UIC), Chicago, USA; 155 The University of Iowa, Iowa City, USA; 156 Johns Hopkins University, Baltimore, USA; 157 The University of Kansas, Lawrence, USA; 158 Kansas State University, Manhattan, USA; 159 Lawrence Livermore National Laboratory, Livermore, USA; 160 University of Maryland, College Park, USA; 161 Massachusetts Institute of Technology, Cambridge, USA; 162 University of Minnesota, Minneapolis, USA; 163 University of Mississippi, Oxford, USA; 164 University of Nebraska-Lincoln, Lincoln, USA; 165 State University of New York at Buffalo, Buffalo, USA; 166 Northeastern University, Boston, USA; 167 Northwestern University, Evanston, USA; 168 University of Notre Dame, Notre Dame, USA; 169 The Ohio State University, Columbus, USA; 170 Princeton University, Princeton, USA; 171 University of Puerto Rico, Mayaguez, USA; 172 Purdue University, West Lafayette, USA; 173 Purdue University Calumet, Hammond, USA; 174 Rice University, Houston, USA; 175 University of Rochester, Rochester, USA; 176 The Rockefeller University, New York, USA; 177 Rutgers, The State University of New Jersey, Piscataway, USA; 178 University of Tennessee, Knoxville, USA; 179 Texas A&M University, College Station, USA; 180 Texas Tech University, Lubbock, USA; 181 Vanderbilt University, Nashville, USA; 182 University of Virginia, Charlottesville, USA; 183 Wayne State University, Detroit, USA; 184 University of Wisconsin, Madison, USA

## Abstract

A search for a standard-model-like Higgs boson in the H→WW and H→ZZ decay channels is reported, for Higgs boson masses in the range 145<*m*_H_<1000 GeV. The search is based upon proton–proton collision data samples corresponding to an integrated luminosity of up to 5.1 fb^−1^ at $\sqrt{s} = 7~\mbox{TeV}$ and up to 5.3 fb^−1^ at $\sqrt{s} = 8~\mbox{TeV}$, recorded by the CMS experiment at the LHC. The combined upper limits at 95 % confidence level on products of the cross section and branching fractions exclude a standard-model-like Higgs boson in the range 145<*m*_H_<710 GeV, thus extending the mass region excluded by CMS from 127–600 GeV up to 710 GeV.

## Introduction

The standard model (SM) of electroweak interactions [1–3] relies on the existence of the Higgs boson, H, a scalar particle associated with the field responsible for spontaneous electroweak symmetry breaking [4–9]. The mass of the boson, *m*_H_, is not predicted by the theory. Searches for the SM Higgs boson at LEP and the Tevatron excluded at 95 % confidence level (CL) masses lower than 114.4 GeV [10] and the mass range 162–166 GeV [11], respectively. Previous direct searches at the Large Hadron Collider (LHC) [12] were based on data from proton–proton (pp) collisions corresponding to an integrated luminosity of up to 5 fb^−1^, collected at a center-of-mass energy $\sqrt{s}=7~\text{TeV}$. Using the 7 TeV data set the Compact Muon Solenoid (CMS) experiment has excluded at 95 % CL masses from 127 to 600 GeV [13]. In 2012, the LHC pp center-of-mass energy was increased to $\sqrt{s}=8~\text{TeV}$, and an additional integrated luminosity of more than 5 fb^−1^ was recorded by the end of June. Searches based on these data in the mass range 110–145 GeV led to the observation of a new boson with a mass of approximately 125 GeV [14–16]. Using this data set the ATLAS experiment excluded at 95 % CL the mass ranges 111–122 and 131–559 GeV [14]. By the end of 2012 the amount of collected integrated luminosity at 8 TeV reached almost 20 fb^−1^. We intend to report findings from the entire data set in a future publication. However, given the heightened interest following the recent discovery of the 125 GeV boson, and the fact that the analysis of the full data taken in 2011–2012 will take time, we present here a search for the SM-like Higgs boson up to 1 TeV with the same data set that was used in Refs. [15, 16].

The observation of a Higgs boson with a mass of 125 GeV is consistent with the theoretical constraint coming from the unitarization of diboson scattering at high energies [17–26]. However, there is still a possibility that the newly discovered particle has no connection to the electroweak symmetry breaking mechanism [27, 28]. In addition, several popular scenarios, such as general two-Higgs-doublet models (for a review see [29, 30]) or models in which the SM Higgs boson mixes with a heavy electroweak singlet [31], predict the existence of additional resonances at high mass, with couplings similar to the SM Higgs boson. In any such models, issues related to the width of the resonance and its interference with non-resonant WW and ZZ backgrounds must be understood. This paper reports a search for a SM-like Higgs boson at high mass, assuming the properties predicted by the SM. The H→WW and H→ZZ decay channels are used as benchmarks for cross section and production mechanism in the mass range 145<*m*_H_<1000 GeV. This approach allows for a self-consistent and coherent presentation of the results at high mass.

For a Higgs boson decaying to two W bosons, the fully leptonic (H→WW→*ℓνℓν*) and semileptonic (H→WW→*ℓν*qq) final states are considered in this analysis. For a Higgs boson decaying into two Z bosons, final states containing four leptons (H→ZZ→2*ℓ*2*ℓ*′), two leptons and two jets (H→ZZ→2*ℓ*2q), and two leptons and two neutrinos (H→ZZ→2*ℓ*2*ν*), are considered, where *ℓ*=e or *μ* and *ℓ*′=e, *μ*, or *τ*. The analyses use pp collision data samples recorded by the CMS detector, corresponding to integrated luminosities of up to 5.1 fb^−1^ at $\sqrt{s} = 7~\text{TeV}$ and up to 5.3 fb^−1^ at $\sqrt{s} = 8~\text{TeV}$.

## The CMS detector and simulations

A full description of the CMS apparatus is available elsewhere [32]. The CMS experiment uses a right-handed coordinate system, with the origin at the nominal interaction point, the *x* axis pointing to the center of the LHC ring, the *y* axis pointing up (perpendicular to the plane of the LHC ring), and the *z* axis along the counterclockwise-beam direction. The polar angle *θ* is measured from the positive *z* axis, and the azimuthal angle *ϕ* is measured in the *x*–*y* plane. All angles in this paper are presented in radians. The pseudorapidity is defined as *η*=−ln[tan(*θ*/2)].

The central feature of the CMS apparatus is a superconducting solenoid of 6  m internal diameter, which provides a magnetic field of 3.8  T. Within the field volume are a silicon pixel and strip tracker, a lead tungstate crystal electromagnetic calorimeter (ECAL), and a brass/scintillator hadron calorimeter. A quartz-fiber Cherenkov calorimeter extends the coverage to |*η*|<5.0. Muons are measured in gas-ionization detectors embedded in the steel flux return yoke. The first level of the CMS trigger system, composed of custom hardware processors, is designed to select the most interesting events in less than 3 μs, using information from the calorimeters and muon detectors. The high level trigger processor farm decreases the event rate from 100  kHz delivered by the first level trigger to a few hundred hertz, before data storage.

Several Monte Carlo (MC) event generators are used to simulate the signal and background event samples. The H→WW and H→ZZ signals are simulated using the next-to-leading order (NLO) package powheg [33–35]. The Higgs boson signals from gluon fusion (gg→H), and vector-boson fusion (VBF, qq→qqH), are generated with powheg at NLO and a dedicated program [36] used for angular correlations. Samples of WH, ZH, and $\mathrm{t}\overline{\mathrm{t}} \mathrm{H}$ events are generated using pythia 6.424 [37].

At generator level, events are weighted according to the total cross section *σ*(pp→H), which contains contributions from gluon fusion computed to next-to-next-to-leading order (NNLO) and next-to-next-to-leading-log (NNLL) [38–49], and from weak-boson fusion computed at NNLO [41, 50–54].

The simulated WW(ZZ) invariant mass *m*_WW_ (*m*_ZZ_) lineshape is corrected to match the results presented in Refs. [55–57], where the complex-pole scheme for the Higgs boson propagator is used. In the gluon fusion production channel, the effects on the lineshape due to interference between Higgs boson signal and the gg→WW and gg→ZZ backgrounds are included [58, 59]. The theoretical uncertainties on the lineshape due to missing higher-order corrections in the interference between background and signal are included in the total uncertainties, in addition to uncertainties associated with electroweak corrections [56, 58]. Interference outside the Higgs boson mass peak has sizable effects on the normalization for those final states where the Higgs boson invariant mass cannot be fully reconstructed. A correction is applied, taking into account the corresponding theoretical uncertainties, in the WW→*ℓν*qq final state [58, 59]. In the WW→*ℓνℓν* and ZZ→2*ℓ*2*ν* final states, the effect of interference on the normalization, as computed in [59, 60], is included with an associated uncertainty of 100 %.

The background contribution from $\mathrm{q} \overline{\mathrm{q}} \to \mathrm{WW} $ production is generated using the MadGraph package [61], and the subdominant gg→WW process is generated using gg2ww [62]. The $\mathrm{q} \overline{\mathrm{q}} \to \mathrm{ZZ} $ production process is simulated at NLO with powheg, and the gg→ZZ process is simulated using gg2zz [63]. Other diboson processes (WZ, Z*γ* ^(∗)^, W*γ* ^(∗)^) and Z+jet are generated with pythia 6.424 and MadGraph. The $\mathrm{t}\overline{\mathrm{t}} $ and tW events are generated at NLO with powheg. For all samples pythia is used for parton showering, hadronization, and underlying event simulation. For leading-order (LO) generators, the default set of parton distribution functions (PDF) used to produce these samples is CTEQ6L [64], while CT10 [65] is used for NLO generators. The *τ*-lepton decays are simulated with tauola [66]. The detector response is simulated using a detailed description of the CMS detector, based on the Geant4 package [67], with event reconstruction performed identically to that for recorded data. The simulated samples include the effect of multiple pp interactions per bunch crossing (pileup). The pythia parameters for the underlying events and pileup interactions are set to the Z2 (Z2^∗^) tune for the 7 (8) TeV data sample as described in Ref. [68] with the pileup multiplicity distribution matching that seen in data.

## Event reconstruction

A complete reconstruction of the individual particles emerging from each collision event is obtained via a particle-flow (PF) technique [69, 70]. This approach uses the information from all CMS sub-detectors to identify and reconstruct individual particles in the collision event, classifying them into mutually exclusive categories: charged hadrons, neutral hadrons, photons, electrons, and muons.

The electron reconstruction algorithm combines information from clusters of energy deposits in the ECAL with the trajectory in the inner tracker [71, 72]. Trajectories in the tracker volume are reconstructed using a dedicated model of electron energy loss, and fitted with a Gaussian sum filter. Electron identification relies on a multivariate (MVA) technique that combines observables sensitive to the amount of bremsstrahlung along the electron trajectory, the geometrical and momentum matching between the electron trajectory and the associated clusters, and shower-shape observables.

The muon reconstruction algorithm combines information from the silicon tracker and the muon spectrometer. Muons are selected from amongst the reconstructed muon-track candidates by applying requirements on the track components in the muon system and on matched energy deposits in the calorimeters [73].

The *τ*-leptons are identified in both the leptonic decay modes, with an electron or muon as measurable decay product, and in the hadronic mode (denoted *τ*_h_). The PF particles are used to reconstruct *τ*_h_ using the “hadron-plus-strip” (HPS) algorithm [74].

Jets are reconstructed from PF candidates by using the anti-*k*_T_ clustering algorithm [75, 76] with a distance parameter of 0.5. Jet energy corrections are applied to account for the non-linear response of the calorimeters, and other instrumental effects. These corrections are based on in-situ calibration using dijet and *γ*/Z+jet data samples [77]. The median energy density due to pileup is evaluated in each event, and the corresponding energy is subtracted from each jet [78]. Jets are required to originate at the primary vertex, which is identified as the vertex with the highest summed $p_{\mathrm{T}} ^{2}$ of its associated tracks. Jets displaced from the primary vertex in the transverse direction can be tagged as b jets [79].

Charged leptons from W and Z boson decays are typically expected to be isolated from other activity in the event. The isolation of e or *μ* leptons is therefore ensured by applying requirements on the sum of the transverse energies of all reconstructed particles, charged or neutral, within a cone of $\Delta R = \sqrt{(\Delta\eta)^{2} + (\Delta\phi)^{2}} < 0.4$ around the lepton direction, after subtracting the average pileup energy estimated using a “jet area” technique [80] on an event-by-event basis.

The magnitude of the transverse momentum (*p*_T_) is calculated as $p_{\mathrm{T}} = \sqrt{\smash[b]{p_{x}}^{2} + \smash[b]{p_{y}}^{2}}$. The missing transverse energy vector $\boldsymbol{E}_{\mathrm{T}}^{\text {miss}}$ is defined as the negative vector sum of the transverse momenta of all reconstructed particles in the event, with $E_{\mathrm{T}}^{\text{miss}} = |\boldsymbol{E}_{\mathrm{T}}^{\text {miss}}|$.

At trigger level, depending on the decay channel, events are required to have a pair of electrons or muons, or an electron and a muon, one lepton with *p*_T_>17 GeV and the other with *p*_T_>8 GeV, or a single electron (muon) with *p*_T_>27 (24) GeV.

The efficiencies for trigger selection, reconstruction, identification, and isolation of e and *μ* are measured from recorded data, using a “tag-and-probe” [81] technique based on an inclusive sample of Z-boson candidate events. These measurements are performed in several bins of $p_{\mathrm{T}} ^{\ell} $ and |*η* ^*ℓ*^|. The overall trigger efficiency for events selected for this analysis ranges from 96 % to 99 %. The efficiency of the electron identification in the ECAL barrel (endcaps) varies from around 82 % (73 %) at $p_{\mathrm{T}} ^{\mathrm{e}} \simeq10~\mbox {GeV}$ to 90 % (89 %) for $p_{\mathrm{T}} ^{\mathrm{e}} \simeq 20~\mbox{GeV}$. It drops to about 85 % in the transition region, 1.44<|*η* ^e^|<1.57, between the ECAL barrel and endcaps. Muons with *p*_T_>5 GeV are reconstructed and identified with efficiencies greater than ∼98 % in the full |*η* ^*μ*^|<2.4 range. The efficiency of the *τ*_h_ identification is around 50 % for $p_{\mathrm{T}} ^{\tau}> 20~\mbox{GeV}$ [74].

## Data analysis

The results presented in this paper are obtained by combining Higgs boson searches exploiting different production and decay modes. A summary of these searches is given in Table 1. All final states are exclusive, with no overlap between channels. The results of the searches in the mass range *m*_H_<145 GeV are presented in Refs. [15, 16]. The presence of a signal in any one of the channels, at a certain value of the Higgs boson mass, is expected to manifest itself as an excess extending around that value for a range corresponding to the Higgs boson width convoluted with the experimental mass resolution. The Higgs boson width varies from few percents of *m*_H_ at low masses through up to 50 % at *m*_H_=1 TeV. The mass resolution for each decay mode is given in Table 1. It should be noted that the presence of the boson with *m*_H_=125 GeV effectively constitutes an additional background especially in the WW→*ℓνℓν* channel up to approximately *m*_H_=200 GeV, because of the poor mass resolution of this analysis. To take this effect explicitly into account a simulated SM Higgs boson signal with *m*_H_=125 GeV is considered as background in this paper.

**Table 1:** Summary information on the analyses included in this paper. The column “H production” indicates the production mechanism targeted by an analysis; it does not imply 100 % purity. The main contribution in the untagged and inclusive categories is always gluon fusion. The (jj)_VBF_ refers to dijet pair consistent with the VBF topology, and (jj)_W(Z)_ to a dijet pair with an invariant mass consistent with coming from a W (Z) dijet decay. For the WW→*ℓνℓν* and ZZ→2*ℓ*2*ℓ*′ channels the full possible mass range starts from 110 GeV, but in this paper both analyses are restricted to the masses above 145 GeV. The ZZ→2*ℓ*2q analysis uses only 7 TeV data. The notation “((ee,*μμ*),e*μ*)+(0 or 1 jets)” indicates that the analysis is performed in two independent lepton categories (ee,*μμ*) and (e*μ*), each category further subdivided in two subcategories with zero or one jets, thus giving a total of four independent channels

H decay mode	H production	Exclusive final states	No. of channels	*m*_H_ range [GeV]	*m*_H_ resolution
WW→*ℓνℓν*	0/1-jets	((ee,*μμ*),e*μ*)+(0 or 1 jets)	4	145–600	20 %
WW→*ℓνℓν*	VBF tag	((ee,*μμ*),e*μ*)+(jj)_VBF_	2	145–600	20 %
WW→*ℓν*qq	Untagged	(e*ν*,*μν*)+((*jj*)_W_ with 0 or 1 jets)	4	180–600	5–15 %
ZZ→2*ℓ*2*ℓ*′	Inclusive	4e, 4*μ*, 2e2*μ*	3	145–1000	1–2 %
		(ee,*μμ*)+(*τ*_h_ *τ*_h_,*τ*_e_ *τ*_h_,*τ*_*μ*_ *τ*_h_,*τ*_e_ *τ*_*μ*_)	8	200–1000	10–15 %
ZZ→2*ℓ*2q	Inclusive	(ee,*μμ*)+((jj)_Z_ with 0, 1, 2b-tags)	6	200–600	3 %
ZZ→2*ℓ*2*ν*	Untagged	(ee,*μμ*)+0, 1, 2 non-VBF jets	6	200–1000	7 %
ZZ→2*ℓ*2*ν*	VBF tag	(ee,*μμ*)+(jj)_VBF_	2	200–1000	7 %

The results of all analyses are finally combined following the prescription developed by the ATLAS and CMS Collaborations in the context of the LHC Higgs Combination Group [82], as described in Ref. [13], taking into account the systematic uncertainties and their correlations.

### H→WW→*ℓνℓν*

In this channel, the Higgs boson decays to two W bosons, both of which decay leptonically, resulting in a signature with two isolated, oppositely charged, high-*p*_T_ leptons (electrons or muons) and large $E_{\mathrm{T}}^{\text{miss}} $ due to the undetected neutrinos. The analysis is very similar to that reported in Refs. [15, 16], but additionally uses an improved Higgs boson mass lineshape model, and uses an MVA shape analysis [83] for data taken at $\sqrt{s}=8~\text{TeV}$. Candidate events must contain two reconstructed leptons with opposite charge, with *p*_T_>20 GeV for the leading lepton, and *p*_T_>10 GeV for the second lepton. Only electrons (muons) with |*η*|<2.5 (2.4) are considered in this channel.

Events are classified into three mutually exclusive categories, according to the number of reconstructed jets with *p*_T_>30 GeV and |*η*|<4.7. The categories are characterized by different signal yields and signal-to-background ratios. In the following these are referred to as 0-jet, 1-jet, and 2-jet samples. Events with more than two jets are considered only if they are consistent with the VBF hypothesis and therefore must not have additional jets in the pseudorapidity region between the highest-*p*_T_ jets. Signal candidates are further divided into same-flavor leptons (e^+^e^−^, *μ* ^+^ *μ* ^−^) and different-flavor leptons (e^±^ *μ* ^∓^) categories. The bulk of the signal arises through direct W decays to electrons or muons, with the small contribution from $\mathrm{W} \to\tau\nu \to\ell\mathrm{+X}$ decays implicitly included. The different-flavor lepton 0-jet and 1-jet categories are analysed with a multivariate technique, while all others make use of sequential selections.

In addition to high-*p*_T_ isolated leptons and minimal jet activity, $E_{\mathrm{T}}^{\text{miss}} $ is expected to be present in signal events, but generally not in background. For this channel, a ${E}_{\text{T, projected}}^{\text{miss}} $ variable is employed. The $E_{\text{T, projected}}^{\text {miss}} $ is defined as (i) the magnitude of the $\boldsymbol{E}_{\mathrm{T}}^{\text{miss}}$ component transverse to the closest lepton, if $\Delta\phi(\ell, \boldsymbol{E}_{\mathrm{T}}^{\text{miss}}) < \pi/2$, or (ii) the magnitude of the $\boldsymbol{E}_{\mathrm{T}}^{\text {miss}}$ otherwise. This observable more efficiently rejects Z/*γ* ^∗^→*τ* ^+^ *τ* ^−^ background events in which the $\boldsymbol{E}_{\mathrm{T}}^{\text{miss}}$ is preferentially aligned with the leptons, and $\mathrm{Z}/ \gamma ^{*}\mathrm{\to\ell ^{+}\ell^{-}} $ events with mismeasured $\boldsymbol{E}_{\mathrm{T}}^{\text{miss}}$. Since the $E_{\text{T, projected}}^{\text {miss}} $ resolution is degraded as pileup increases, the minimum of two different observables is used: the first includes all particle candidates in the event, while the second uses only the charged particle candidates associated with the primary vertex. Events with $E_{\text{T, projected}}^{\text {miss}} $ above 20 GeV are selected for this analysis.

The backgrounds are suppressed using techniques described in Refs. [15, 16]. Top quark background is controlled with a top-quark-tagging technique based on soft muon and b-jet tagging [79]. A minimum dilepton transverse momentum ($p_{\mathrm{T}} ^{\ell\ell}$) of 45 GeV is required, in order to reduce the W+jets background. Rejection of events with a third lepton passing the same requirements as the two selected leptons reduces both WZ and W*γ* ^∗^ backgrounds. The background from low-mass resonances is rejected by requiring a dilepton mass *m*_*ℓℓ*_>12 GeV.

The Drell–Yan process produces same-flavor lepton pairs (e^+^e^−^ and *μ* ^+^ *μ* ^−^) and therefore additional requirements are applied for the same-flavor final state. Firstly, the resonant component of the Drell–Yan background is rejected by requiring a dilepton mass outside a 30 GeV window centered on the Z-boson mass. The remaining off-peak contribution is further suppressed by requiring $E_{\text{T, projected}}^{\text {miss}} >45~\mbox{GeV}$. For events with two jets, the dominant source of misreconstructed $E_{\mathrm{T}}^{\text{miss}}$ is the mismeasurement of the hadronic recoil, and optimal performance is obtained by requiring $E_{\mathrm {T}}^{\text{miss}} >45~\mbox{GeV}$. Finally, the momenta of the dilepton system and of the most energetic jet must not be back-to-back in the transverse plane. These selections reduce the Drell–Yan background by three orders of magnitude, while rejecting less than 50 % of the signal.

**Fig. 1:**
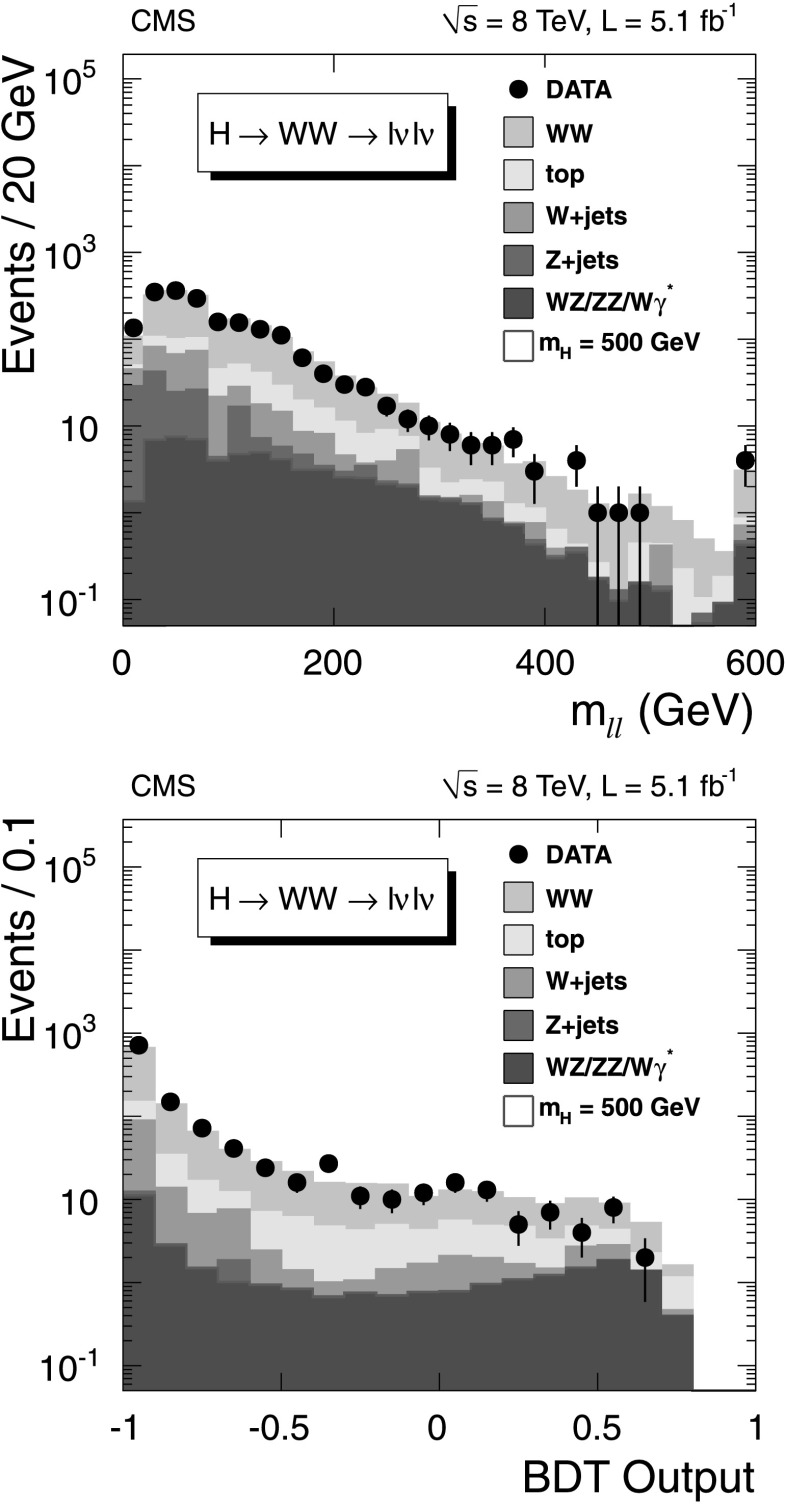
(*Top*) Distributions of *m*_*ℓℓ*_ in the 0-jet different-flavor category of the WW→*ℓνℓν* channel for data (*points* with *error bars*), for the main backgrounds (*stacked histograms*), and for a SM Higgs boson signal with *m*_H_=500 GeV. The standard preselection is applied. (*Bottom*) BDT-classifier distributions for signal and background events for a SM Higgs boson with *m*_H_=500 GeV and for the main backgrounds in the 0-jet different-flavor category after requiring $80 < m_{\mathrm{T}} ^{\ell\ell , E_{\mathrm{T}}^{\text{miss}} } < 500~\mbox {GeV}$ and *m*_*ℓℓ*_<500 GeV

These requirements form the set of “preselection” criteria. The preselected sample is dominated by non-resonant WW events. Figure 1(top) shows an example of the *m*_*ℓℓ*_ distribution for the 0-jet different-flavor-leptons category after the preselection. The data are well reproduced by the simulation. To enhance the signal-to-background ratio, loose *m*_H_-dependent requirements are applied on *m*_*ℓℓ*_ and the transverse mass, given by:

$$ m_\mathrm{T} ^{\ell\ell, E_{\mathrm {T}}^{\text{miss}} } = \sqrt{2 p_{\mathrm {T}} ^{\ell\ell} E_{\mathrm{T}}^{\text{miss}} (1-\cos \Delta\phi_{ \ell \ell , E_{\mathrm{T}}^{\text{miss}} } )}, $$

where $\Delta\phi_{ \ell \ell , E_{\mathrm{T}}^{\text{miss}} } $ is the difference in azimuth between $\boldsymbol {E}_{\mathrm{T}}^{\text{miss}}$ and $\boldsymbol{p}_{\mathrm{T}}^{\ell\ell} $. After preselection, a multivariate technique is employed for the different-flavor final state in the 0-jet and 1-jet categories. In this approach, a boosted decision tree (BDT) [84] is trained for each Higgs boson mass hypothesis and jet category to discriminate signal from background.

The multivariate technique employs the variables used in the preselection and additional observables including Δ*R*_*ℓℓ*_ between the leptons and the $m_{\mathrm{T}} ^{\ell \ell, E_{\mathrm{T}}^{\text{miss}} }$. For the 1-jet category the $\Delta\phi_{ \ell \ell , E_{\mathrm{T}}^{\text{miss}} } $ and azimuthal angle between the $\boldsymbol{p}_{\mathrm {T}}^{\ell\ell} $ and the jet are also used. The BDT classifier distributions for *m*_H_=500 GeV are shown in Fig. 1 (bottom) for the 0-jet different-flavor category. BDT training is performed using H→WW as signal and non-resonant WW as background. The sum of templates for the signal and background are fitted to the binned observed BDT distributions.

The 2-jet category is optimized for the VBF production mode [50, 51, 53, 85], for which the cross section is roughly ten times smaller than for the gluon fusion mode. Sequential selections are employed for this category. The main requirements for selecting the VBF-type events are on the mass of the dijet system, *m*_jj_>450 GeV, and on the angular separation of the two jets |Δ*η*_jj_|>3.5. An *m*_H_-dependent requirement on the dilepton mass is imposed, as well as other selection requirements that are independent of the Higgs boson mass hypothesis.

The normalization of the background contributions relies on data whenever possible and exploits a combination of techniques [15, 16]. The $\mathrm{t}\overline{\mathrm{t}} $ background is estimated by extrapolation from the observed number of events with the b-tagging requirement inverted. The Drell–Yan background measurement is based on extrapolation from the observed number of e^+^e^−^, *μ* ^+^ *μ* ^−^ events with the Z-veto requirement inverted. The background of W+jets and QCD multi-jet events is estimated by measuring the number of events with one lepton passing a loose requirement on isolation. The probability for such loosely-isolated non-genuine leptons to pass the tight isolation criteria is measured in data using multi-jet events. The non-resonant WW contribution is estimated from simulation.

Experimental effects, theoretical predictions, and the choice of event generators are considered as sources of systematic uncertainty, and their impact on the signal efficiency is assessed. The impact on the kinematic distributions is also considered for the BDT analysis. The overall signal yield uncertainty is estimated to be about 20 %, and is dominated by the theoretical uncertainty associated with missing higher-order QCD corrections and PDF uncertainties, estimated following the PDF4LHC recommendations [86–90]. The total uncertainty on the background estimation in the H→WW signal region is about 15 % and is dominated by the statistical uncertainty on the observed number of events in the background control regions.

**Fig. 2:**
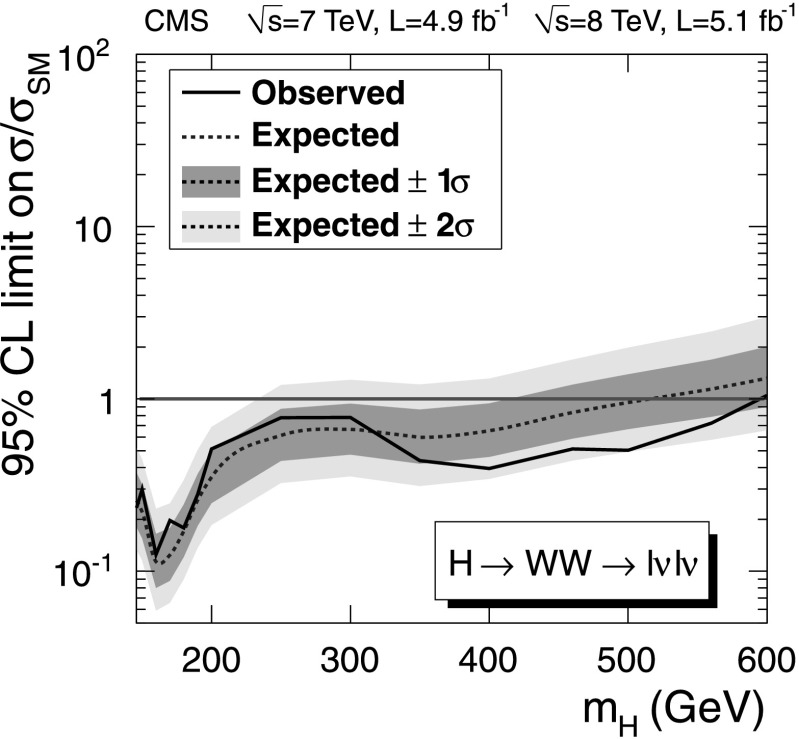
Observed (*solid line*) and expected (*dashed line*) 95 % CL upper limit on the ratio of the product of production cross section and branching ratio to the SM expectation for the Higgs boson obtained using the asymptotic CL_S_ technique [91, 92] in the WW→*ℓνℓν* channel. The 68 % (1*σ*) and 95 % (2*σ*) CL ranges of expectation for the background-only model are also shown with *green* and *yellow bands*, respectively. The *horizontal solid line* at unity indicates the SM expectation (Color figure online)

After applying the final selections, no evidence of a SM-like Higgs boson is observed over the mass range considered in this paper. Upper limits are derived on the ratio of the product of the Higgs boson production cross section and the H→WW branching fraction, $\sigma_{\mathrm{H}} \times\mathcal{B}(\mathrm{H}\to \mathrm{WW} )$, to the SM expectation. The observed and expected upper limits at 95 % confidence level (CL) with all categories combined are shown in Fig. 2. The contribution of the 2-jet category to the expected limits is approximately 10 %.

### H→WW→*ℓν*qq

The WW semileptonic channel has the largest branching fraction of all the channels presented in this paper. Its advantage over the fully leptonic final state is that it has a reconstructable Higgs boson mass peak [93]. This comes at the price of a large W+jets background. The level to which this background can be controlled largely determines the sensitivity of the analysis. This is the first time CMS is presenting a measurement in this decay channel.

The reconstructed electrons (muons) are required to have *p*_T_>35 (25) GeV, and are restricted to |*η*|<2.5 (2.1). The jets are required to have *p*_T_>30 GeV and |*η*|<2.4, and not to overlap with the leptons, with the overlap determined by a cone around the lepton axis of radius Δ*R*=0.3. Events with electrons and muons, and with exactly two or three jets are analysed separately, giving four categories in total. The two highest-*p*_T_ jets are assumed to arise from the hadronic decay of the W candidate. According to simulation, in the case of 2 (3) jet events, the correct jet-combination rate varies from 68 (26) % for *m*_H_=200 GeV to 88 (84) % for *m*_H_=600 GeV. For low *m*_H_ values jets produced in initial or final state radiation are often more energetic than jets from W decay, therefore in 3 jet events the correct jet-combination rate decreases quickly with decreasing *m*_H_. Events with an incorrect dijet combination result in a broad non-peaking background in the *m*_WW_ spectrum.

The leptonic W candidate is reconstructed from the $(\ell, E_{\mathrm{T}}^{\text{miss}} )$ system. Events are required to have $E_{\mathrm {T}}^{\text{miss}} > 30~(25)~\mbox{GeV}$ for the electron (muon) categories. To reduce the background from processes that do not contain W→*ℓν* decays, requirements of $m_{\mathrm{T}}^{\ell, E_{\mathrm{T}}^{\text {miss}} }>30~\mbox{GeV}$ and $\lvert\Delta\phi_{\textrm{leading jet,$E_{\mathrm{T}}^{\text{miss}}$}} \rvert > 0.8~(0.4)$ are imposed for electrons (muons). The $m_{\mathrm{T}}^{\ell , E_{\mathrm{T}}^{\text{miss}} }$ is defined as

$$ m_\mathrm{T} ^{\ell, E_{\mathrm {T}}^{\text{miss}} } = \sqrt{2 p_{\mathrm {T}} ^{\ell} E_{\mathrm{T}}^{\text{miss}} (1-\cos \Delta\phi_{ \ell , E_{\mathrm {T}}^{\text{miss}} } )}, $$

where $\Delta\phi_{ \ell , E_{\mathrm{T}}^{\text{miss}} } $ is the difference in azimuth between $\boldsymbol {E}_{\mathrm{T}}^{\text{miss}}$ and $\boldsymbol {p}_{\mathrm{T}}^{\ell} $. These criteria reduce the QCD multijet background, for which in many cases the $E_{\mathrm{T}}^{\text{miss}} $ is generated by a mismeasurement of a jet energy.

To improve the *m*_WW_ resolution, both W candidates are constrained in a kinematic fit to the W-boson mass to within its known width. For the W→qq candidate the fit uses the four-momenta of the two highest-*p*_T_ jets. For the W→*ℓν* candidate the $E_{\mathrm{T}}^{\text{miss}} $ defines the transverse energy of the neutrino and the longitudinal component of the neutrino momentum, *p*_*z*_, is unknown. The ambiguity is resolved by taking the solution that yields the smaller |*p*_*z*_| value for the neutrino. According to simulation over 85 % of signal events receive a correct |*p*_*z*_| value, thus improving the mass resolution, especially at low *m*_H_.

To exploit the differences in kinematics between signal and background events, a likelihood discriminant is constructed that incorporates a set of variables that best distinguishes the Higgs boson signal from the W+jets background. These variables comprise five angles between the Higgs boson decay products, that describe the Higgs boson production kinematics [36]; the *p*_T_ and rapidity of the WW system; and the lepton charge. The likelihood discriminant is optimized with dedicated simulation samples for several discrete Higgs boson mass hypotheses, for each lepton flavor (e, *μ*) and for each jet multiplicity (2-jet, 3-jet) independently. Four different optimizations are therefore obtained per mass hypothesis. For each of them, events are retained if they survive a simple selection on the likelihood discriminant, chosen in order to optimize the expected limit for the Higgs boson production cross section.

To simultaneously extract the relative normalizations of all background components in the signal region, an unbinned maximum likelihood fit is performed on the invariant mass distribution of the dijet system, *m*_jj_. The fit is performed independently for each Higgs boson mass hypothesis. The signal region corresponding to the W mass window, 65<*m*_jj_<95 GeV, is excluded from the fit. The mass window corresponds to approximately twice the dijet mass resolution. The shape of the *m*_jj_ distribution for the W+jets background is determined by simulation. The overall normalization of the W+jets component is allowed to vary in the fit. The shapes for other backgrounds (electroweak diboson, $\mathrm{t}\overline {\mathrm{t}}$, single top quark, and Drell–Yan plus jets) are based on simulation, and their normalizations are constrained to theoretical predictions, within the corresponding uncertainties. The multijet background normalization is estimated from data by relaxing lepton isolation and identification requirements. Its contribution to the total number of events is evaluated from a separate two-component likelihood fit to the $m_{\mathrm{T}}^{\ell, E_{\mathrm{T}}^{\text{miss}} }$ distribution, and constrained in the *m*_jj_ fit according to this fraction within uncertainties. For electrons, the multijet fraction accounts for several percent of the event sample, depending on the number of jets in the event, while for muons it is negligible.

**Fig. 3:**
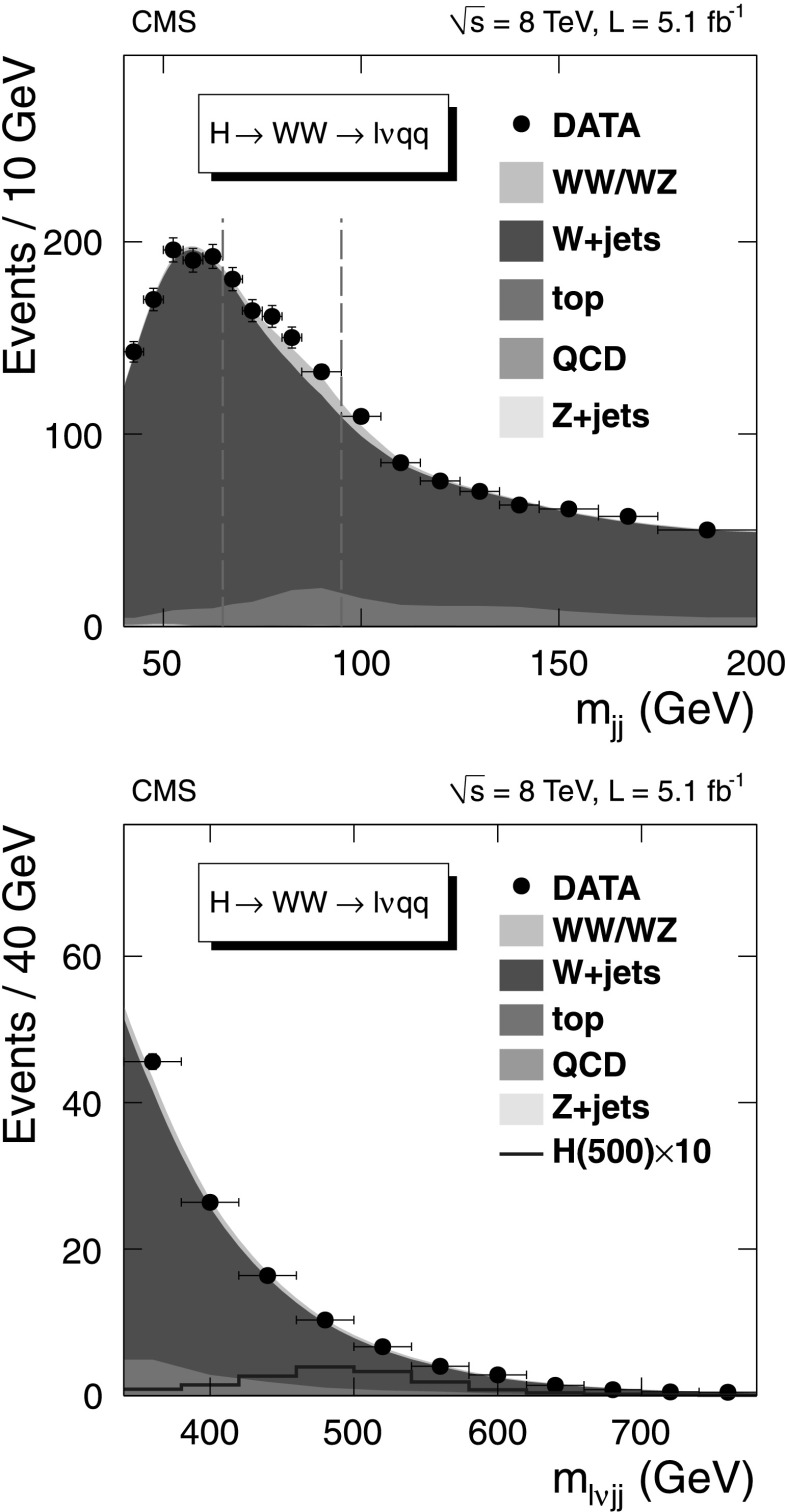
Invariant mass distributions for the *m*_H_=500 GeV mass hypothesis, (*μ*, 2 jets) category in the H→WW→*ℓν*qq channel. (*Top*) The dijet invariant mass distribution with the major background contributions. The *vertical lines* correspond to the signal region of this analysis 65<*m*_jj_<95 GeV. (*Bottom*) The WW invariant mass distribution with the major background contributions in the signal region

Limits are established based on the measured invariant mass of the WW system, *m*_*ℓν*jj_. The *m*_*ℓν*jj_ shape for the major background, W+jets, is extracted from data as a linear combination of the shapes measured in two signal-free sideband regions of *m*_jj_ (55<*m*_jj_<65 GeV, 95<*m*_jj_<115 GeV). The relative fraction of the two sidebands is determined through simulation, separately for each Higgs boson mass hypothesis, by minimizing the *χ* ^2^ between the interpolated *m*_*ℓν*jj_ shape in the signal region and the expected one. The *m*_*ℓν*jj_ shape for multijet background events is obtained from data with the procedure described above. All other background categories use the *m*_*ℓν*jj_ shape from simulation. The *m*_jj_ and *m*_*ℓν*jj_ distributions with final background estimates are shown in Fig. 3, with selections optimized for a 500 GeV Higgs boson mass hypothesis, for the (*μ*, 2 jets) category. The final background *m*_*ℓν*jj_ distribution is obtained by summing up all the individual contributions and smoothing it with an exponential function. The shapes of the *m*_*ℓν*jj_ distribution for total background, signal and data for each mass hypothesis and event category are binned, with bin size approximately equal to the mass resolution, and fed as input to the limit-setting procedure.

The largest source of systematic uncertainty on the background is due to the uncertainty in the shape of the *m*_*ℓν*jj_ distribution of the total background. The shape uncertainty is derived by varying the parameters of the exponential fit function up and down by one standard deviation. The only other uncertainty assigned to background is the normalization uncertainty from the *m*_jj_ fit. Both of these uncertainties are estimated from data. The dominant systematic uncertainties on the signal include theoretical uncertainties for the cross section (14–19 % for gluon fusion) [41] and on jet energy scale (4–28 %), as well as the efficiency of the likelihood selection (10 %). The latter effect is computed by taking the relative difference in efficiency between data and simulation using a control sample of top-quark pair events in data. These events are good proxies for the signal, since in both cases the primary production mechanism is gluon fusion, and the semi-leptonic final states contain decays of two W bosons.

**Fig. 4:**
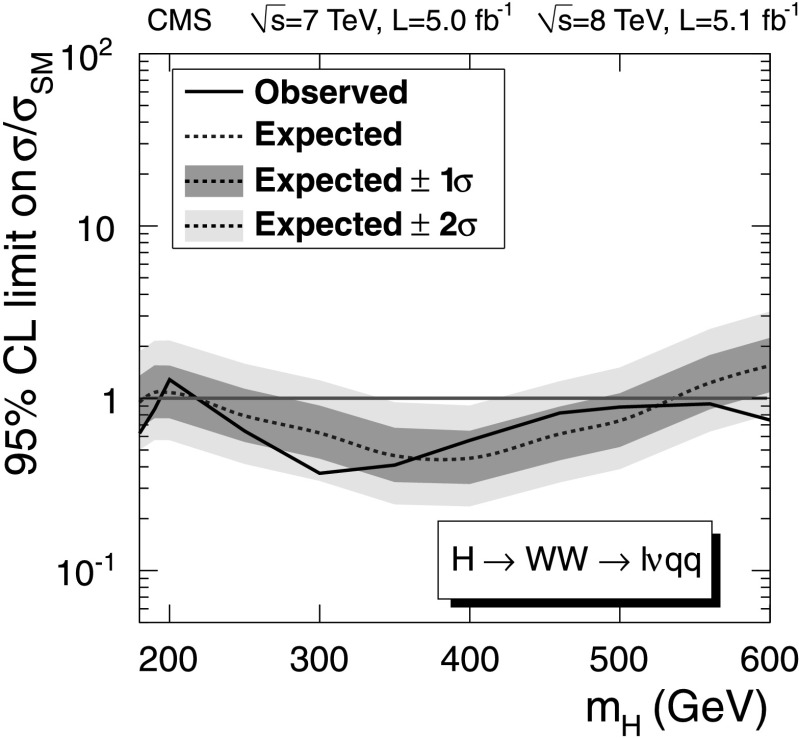
Observed (*solid line*) and expected (*dashed line*) 95 % CL upper limit on the ratio of the product of production cross section and branching fraction to the SM expectation for the Higgs boson in the WW semileptonic channel

The upper limits on the ratio of the production cross section for the Higgs boson compared to the SM expectation are presented in Fig. 4.

### H→ZZ→2*ℓ*2*ℓ*′

This analysis seeks to identify Higgs boson decays to a pair of Z bosons, with both decaying to a pair of leptons. This channel has extremely low background, and the presence of four leptons in the final state allows reconstruction and isolation requirements to be loose. Due to very good mass resolution and high efficiency of the selection requirements, this channel is one of the major discovery channels at both low and high Higgs boson masses. A detailed description of this analysis may be found in [15, 16, 94, 95].

Events included in the analysis contain Z candidates formed from a pair of leptons of the same flavor and opposite charge. Electrons (muons, *τ*_h_) are required to be isolated, to originate from the primary vertex, and to have *p*_T_>7 (5,20) GeV and |*η*|<2.5 (2.1,2.3). The event selection procedure results in mutually exclusive sets of Z candidates in the H→2*ℓ*2*ℓ* and H→2*ℓ*2*τ* channels, with the former identified first.

For the 2*ℓ*2*ℓ* final state, the lepton pair with invariant mass closest to the nominal Z boson mass, denoted Z_1_, is identified and retained if it satisfies $40 < m_{\mathrm{Z}_{1}} < 120~\mbox{GeV}$. The second Z candidate is then constructed from the remaining leptons in the event, and is required to satisfy $12 < m_{\mathrm {Z}_{2}} < 120~\mbox{GeV}$. If more than one Z_2_ candidate remains, the ambiguity is resolved by choosing the leptons of highest *p*_T_. Amongst the four candidate decay leptons, it is required that at least one should have *p*_T_>20 GeV, and that another should have *p*_T_>10 GeV. This requirement ensures that selected events correspond to the high-efficiency plateau of the trigger.

For the 2*ℓ*2*τ* final state, events are required to have one Z_1_→*ℓ* ^+^ *ℓ* ^−^ candidate, with one lepton having *p*_T_>20 GeV and the other *p*_T_>10 GeV, and a Z_2_→*τ* ^+^ *τ* ^−^, with *τ* decaying to *μ*,e or hadrons. The leptons from *τ* leptonic decays are required to have *p*_T_>10 GeV. The invariant mass of the reconstructed Z_1_ is required to satisfy 60<*m*_*ℓℓ*_<120 GeV, and that of the Z_2_ to satisfy *m*_*ττ*_<90 GeV, where *m*_*ττ*_ is the invariant mass of the visible *τ*-decay products.

Simulation is used to evaluate the expected non-resonant ZZ background as a function of *m*_2*ℓ*2*ℓ*′_. The cross section for ZZ production at NLO is calculated with mcfm [96–98]. The theoretical uncertainty on the cross-section is evaluated as a function of *m*_2*ℓ*2*ℓ*′_, by varying the QCD renormalization and factorization scales and the PDF set, following the PDF4LHC recommendations. The uncertainties associated with the QCD and PDF scales for each final state are on average 8 %. The number of predicted ZZ→2*ℓ*2*ℓ*′ events and their associated uncertainties, after the signal selection, are given in Table 2.

**Table 2:** Observed and expected background and signal yields for each final state in the H→ZZ→2*ℓ*2*ℓ*′ channel. For the Z+X background, the estimations are based on data. The uncertainties represent the statistical and systematic uncertainties combined in quadrature

Channel	4e	4*μ*	2e2*μ*	2*ℓ*2*τ*
ZZ background	28.6±3.3	44.6±4.6	70.8±7.5	12.1±1.5
Z+X	$2.3 ^{ + 2.1 }_{ - 1.5 }$	$1.1 ^{ + 0.8 }_{ - 0.7 }$	$3.6 ^{ + 2.9 }_{ - 2.2 }$	8.9±2.5
All backgrounds	$30.9 ^{ + 3.9 }_{ - 3.6 }$	$45.7 ^{ + 4.7 }_{ - 4.7 }$	$74.4 ^{ + 8.0 }_{ - 7.8 }$	21.0±2.9
Observed	26	42	88	20
*m*_H_=350 GeV	5.4±1.4	7.6±1.6	13.2±3.0	3.1±0.8
*m*_H_=500 GeV	1.9±0.9	2.7±1.2	4.6±2.1	1.4±0.7

To allow estimation of the $\mathrm{t}\overline{\mathrm {t}} $, Z+jets, and WZ+jets reducible backgrounds a Z_1_+*ℓ*_ng_ control region is defined, with at least one loosely defined non-genuine lepton candidate, *ℓ*_ng_, in addition to a Z candidate. To avoid possible contamination from WZ events, $E_{\mathrm {T}}^{\text{miss}} < 25~\mbox{GeV}$ is required. This control region is used to determine the misidentification probability for *ℓ*_ng_ to pass the final lepton selections as a function of *p*_T_ and *η*. To estimate the number of expected background events in the signal region, Z_1_+*ℓ* ^±^ *ℓ* ^∓^, this misidentification probability is applied to two control regions, $\mathrm{Z}_{1}+\ell^{\pm}\ell_{\text{ng}}^{\mp}$ and $\mathrm{Z}_{1}+\ell_{\mathrm{ng}}^{\pm}\ell_{\mathrm{ng}}^{\mp}$. The contamination from WZ events containing a genuine additional lepton is suppressed by requiring the imbalance of the measured energy deposition in the transverse plane to be below 25 GeV. The estimated reducible background yield in the signal region is denoted as Z+X in Table 2. The systematic uncertainties associated with the reducible background estimate vary from 30 % to 70 %, and are presented in the table combined in quadrature with the statistical uncertainties.

**Fig. 5:**
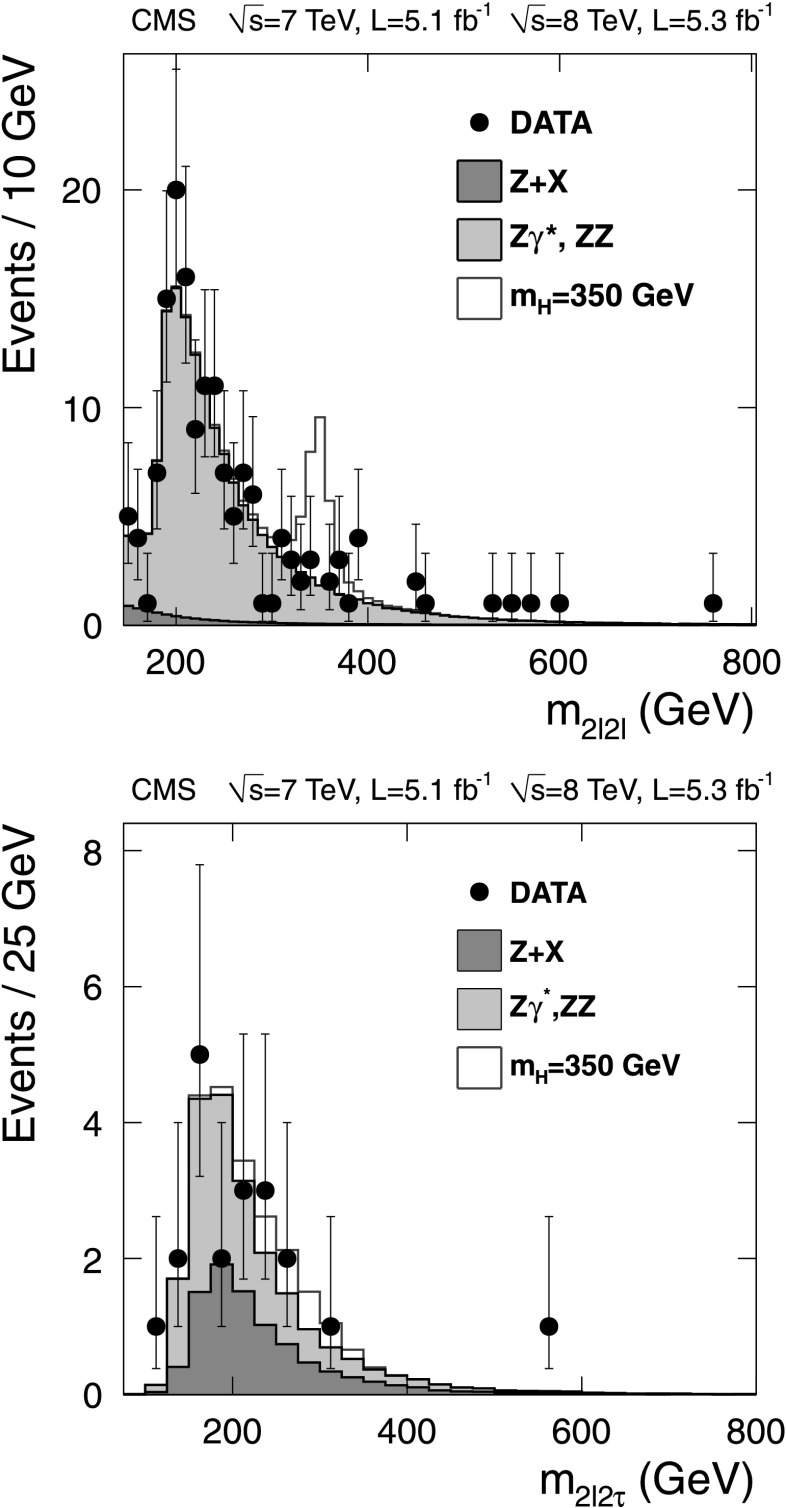
Distribution of the four-lepton reconstructed mass for (*top*) the sum of the 4e, 4*μ*, and 2e2*μ* channels, and for (*bottom*) the sum over all 2*ℓ*2*τ* channels. Points represent the data, *shaded* histograms represent the background, and unshaded histogram the signal expectations. The reconstructed masses in 2*ℓ*2*τ* states are shifted downwards with respect to the true masses by about 30 % due to the undetected neutrinos in *τ* decays

The reconstructed invariant mass distributions for 2*ℓ*2*ℓ*′ are shown in Fig. 5 for the combination of the 4e, 4*μ*, and 2e2*μ* final states in the top plot and for the combination of the 2*ℓ*2*τ* states in the bottom one. The data are compared with the expectation from SM background processes. The observed mass distributions are consistent with the SM background expectation.

**Fig. 6:**
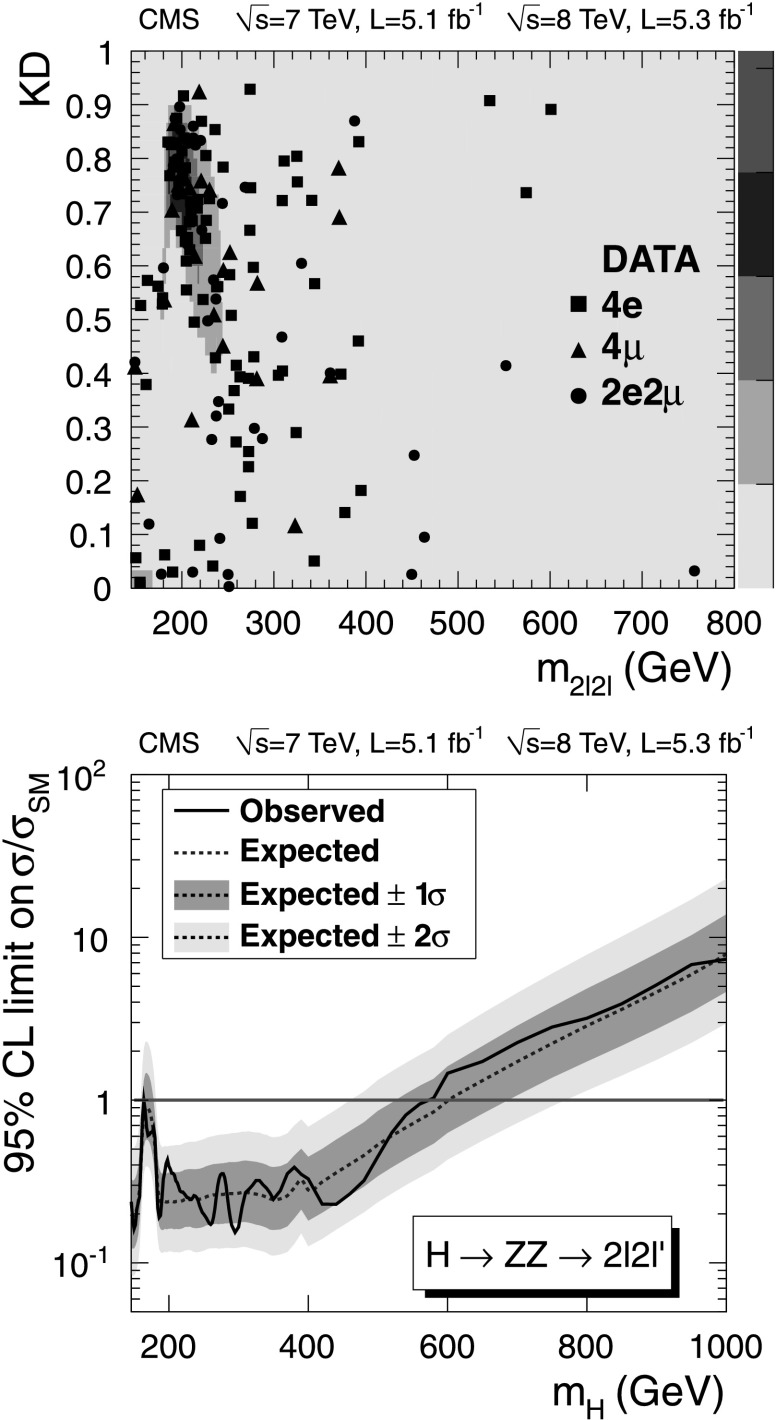
(*Top*) The distribution of events selected in the 2*ℓ*2*ℓ* subchannels for the kinematic discriminant, KD, versus *m*_2*ℓ*2*ℓ*_. Events in the three final states are marked by filled symbols (defined in the legend). The *colored contours* (with the measure on the color scale of the *right axis*) represent the expected relative density of background events. (*Bottom*) Observed (*solid line*) and expected (*dashed line*) 95 % CL upper limits on the ratio of the product of the production cross section and branching fraction to the SM expectation in the H→ZZ→2*ℓ*2*ℓ*′ channel. The 68 % (1*σ*) and 95 % (2*σ*) ranges of expectation for the background-only model are also shown with *green* and *yellow bands*, respectively (Color figure online)

The kinematics of the H→ZZ→2*ℓ*2*ℓ* process, for a given invariant mass of the four-lepton system, are fully described at LO by five angles and the invariant masses of the two lepton pairs [36, 99, 100]. A kinematic discriminant (KD), based on these seven variables, is constructed based on the probability ratio of the signal and background hypotheses [101]. The distribution of KD versus *m*_2*ℓ*2*ℓ*_ is shown in Fig. 6 (top) for the selected event sample, and is consistent with the SM background expectation. The two-dimensional KD-*m*_2*ℓ*2*ℓ*_ distribution is used to set upper limits on the cross-section in the 2*ℓ*2*ℓ* channel. For the 2*ℓ*2*τ* final state, limits are set using the *m*_2*ℓ*2*τ*_ distribution. The combined upper limits from all channels are shown in Fig. 6 (bottom).

### H→ZZ→2*ℓ*2q

This channel has the largest branching fraction of all H→ZZ channels considered in this paper, but also a large background contribution from Z+jets production. The hadronically-decaying Z bosons produce quark jets, with a large fraction of heavy quarks compared to the background that is dominated by gluon and light quark jets. This feature allows the use of a heavy-flavor tagging algorithm to enhance the signal with respect to background. The analysis presented here updates the previously published result [101] by the use of the most recent theoretical predictions for the Higgs boson mass lineshape and the correction of a problem in the background description. The measurement in this channel uses the same $\sqrt{s}=7~\text{TeV}$ data set as the published paper [101] and uses the same selection requirements.

Reconstructed electrons and muons are required to have *p*_T_>40 (20) GeV for the highest-*p*_T_ (second-highest-*p*_T_) lepton. Electrons (muons) are required to have |*η*|<2.5(2.4), with the transition region between ECAL barrel and endcap, 1.44<|*η*|<1.57, excluded for electrons. Jets are required to have *p*_T_>30 GeV and |*η*|<2.4. Each pair of oppositely-charged leptons of the same flavor, and each pair of jets, are considered as Z candidates. Background contributions are reduced by requiring 75<*m*_jj_<105 GeV and 70<*m*_*ℓℓ*_<110 GeV.

In order to exploit the different jet composition of signal and background, events are classified into three mutually exclusive categories, according to the number of selected b-tagged jets: 0b-tag, 1b-tag and 2b-tag. An angular likelihood discriminant is used to separate signal-like from background-like events in each category [36]. A “quark-gluon” likelihood discriminant (qgLD), intended to distinguish gluon jets from light-quark jets, is employed for the 0b-tag category, which is expected to be dominated by Z+jets background. A requirement on the qgLD value reduces backgrounds by approximately 40 % without any loss in the signal efficiency. In order to suppress the substantial $\mathrm{t}\overline{\mathrm{t}} $ background in the 2b-tag category, a discriminant *λ* is used. This variable is defined as the ratio of the likelihoods of a hypothesis with $E_{\mathrm{T}}^{\text {miss}} $ equal to the value measured with the PF algorithm, and the null hypothesis $E_{\mathrm {T}}^{\text{miss}} =0~\mbox{GeV}$ [102]. This discriminant provides a measure of whether the event contains genuine missing transverse energy. Events in the 2b-tag category are required to have 2ln*λ*<10. When an event contains multiple Z candidates passing the selection requirements, only the ones with jets in the highest b-tag category are retained for analysis. If multiple candidates are still present, the ones with *m*_jj_ and *m*_*ℓℓ*_ values closest to the Z mass are retained.

The statistical analysis is based on the invariant mass of the Higgs boson candidate, *m*_ZZ_, applying the constraint that the dijet invariant mass is consistent with that of the Z boson. Data containing a Higgs boson signal are expected to show a resonance peak over a continuum background distribution.

The background distributions are estimated from the *m*_jj_ sidebands, defined as 60<*m*_jj_<75 GeV and 105<*m*_jj_<130 GeV. In simulation, the composition and distribution of the dominant backgrounds in the sidebands are observed to be similar to those in the signal region. The distributions derived from data sidebands are measured for each of the three b-tag categories and used to estimate the normalization of the background and its dependence on *m*_ZZ_. The results of the sideband interpolation procedure are in good agreement with the observed distributions in data. In all cases, the dominant backgrounds include Z+jets with either light- or heavy-flavor jets and $\mathrm{t}\overline{\mathrm{t}}$ background, both of which populate the *m*_jj_ signal region and the *m*_jj_ sidebands. The diboson background amounts to less than 5 % of the total in the 0b and 1b-tag categories, and about 10 % in the 2b-tag category. No significant difference is observed between results from data and the background expectation.

The distribution of *m*_ZZ_ for the background is parametrized by an empirical function constructed of a Crystal Ball distribution [103–105] multiplied by a Fermi function, $f( m_{\mathrm{ZZ}} ) = 1/[1+\mathrm {e}^{-( m_{\mathrm{ZZ}} -a)/b}]$, fitted to the shape and with normalization determined from the sidebands. The dominant normalization uncertainty in the background estimation is due to statistical uncertainty of the number of events in the sidebands. The reconstructed signal distribution has two components. The Double Crystal Ball function [103–105] is used to describe the events with well reconstructed Higgs boson decay products. The *m*_ZZ_ spectrum for misreconstructed events is described with a triangle function with linear rising and falling edges, convoluted with Crystal Ball function for better description of the peak and tail regions. The signal reconstruction efficiency and the *m*_ZZ_ distribution are parametrized as a function of *m*_H_. The main uncertainties in the signal *m*_ZZ_ parametrization are due to experimental resolution, which is predominantly due to the uncertainty on the jet energy scale [77]. Uncertainties in b-tagging efficiency are evaluated with a sample of jet events enriched in heavy flavors by requiring a muon to be spatially close to a jet. The uncertainty associated with the qgLD selection efficiency is evaluated using the *γ*+jet sample in data, which predominantly contains light quark jets.

**Fig. 7:**
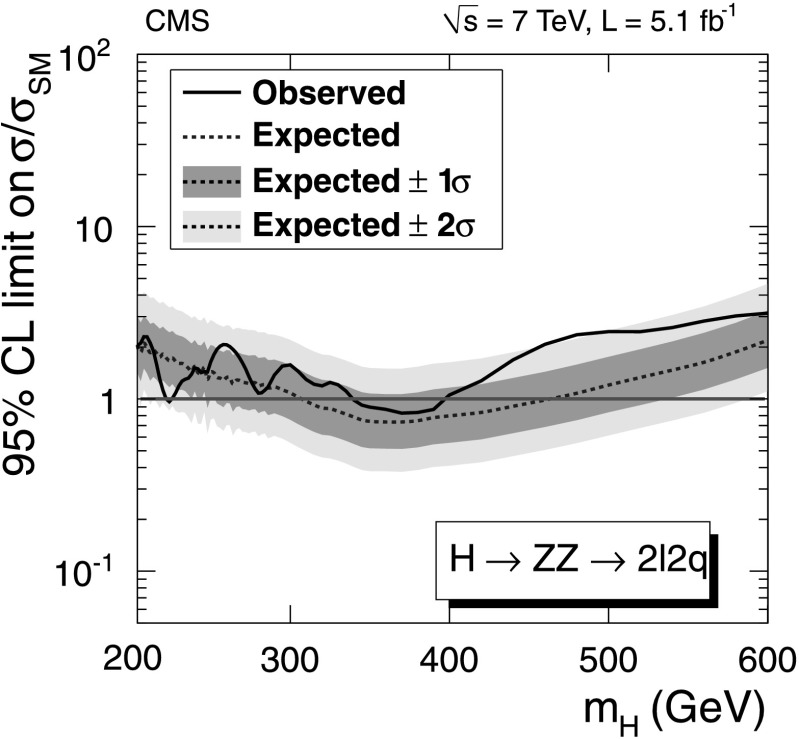
Observed (*solid line*) and expected (*dashed line*) 95 % CL upper limit on the ratio of the product of the production cross section and branching fraction, to the SM expectation for the Higgs boson in the H→ZZ→2*ℓ*2q channel

The upper limits at 95 % CL on the ratio of the production cross section for the Higgs boson to the SM expectation, obtained from the combination of all categories, are presented in Fig. 7. This exclusion limit supersedes the previously published one [101].

### H→ZZ→2*ℓ*2*ν*

This analysis identifies Higgs boson decays to a pair of Z bosons, with one of Z bosons decaying leptonically and the other to neutrinos. A detailed description of the analysis can be found in [106]. The analysis strategy is based on a set of *m*_H_-dependent selection requirements applied on $E_{\mathrm{T}}^{\text{miss}}$ and *m*_T_, where  Events are required to have a pair of well identified, isolated leptons of same flavor (e^+^e^−^ or *μ* ^+^ *μ* ^−^), each with *p*_T_>20 GeV, with an invariant mass within a 30 GeV window centered on the Z mass. The *p*_T_ of the dilepton system is required to be greater than 55 GeV. Jets are considered only if they have *p*_T_>30 GeV and |*η*|<5. The presence of large missing transverse energy in the event is also an essential feature of the signal.

To suppress Z+jets background, events are excluded from the analysis if the angle in the azimuthal plane between the $\boldsymbol {E}_{\mathrm{T}}^{\text{miss}}$ and the closest jet is smaller than 0.5 radians. In order to remove events where the lepton is mismeasured, events are rejected if $E_{\mathrm{T}}^{\text{miss}} > 60~\mbox{GeV}$ and $\Delta\phi(\ell,\boldsymbol{E}_{\mathrm{T}}^{\text {miss}}) < 0.2$. The top-quark background is suppressed by applying a veto on events having a b-tagged jet with *p*_T_>30 GeV and |*η*|<2.4. To further suppress the top-quark background, a veto is applied on events containing a “soft muon”, with *p*_T_>3 GeV, which is typically produced in the leptonic decay of a bottom quark. To reduce the WZ background, in which both bosons decay leptonically, any event with a third lepton (e or *μ*) with *p*_T_>10 GeV, and passing the identification and isolation requirements, is rejected.

The search is carried out in two mutually exclusive categories. The VBF category contains events with at least two jets with |Δ*η*_jj_|>4 and *m*_jj_>500 GeV. Both leptons forming the Z candidate are required to lie in this Δ*η*_jj_ region, and there should be no other jets in it. The gluon fusion category includes all events failing the VBF selection, and is subdivided into subsamples according to the presence or absence of reconstructed jets. The event categories are chosen in order to optimize the expected cross section limit. In the case of the VBF category, a constant $E_{\mathrm{T}}^{\text{miss}} >70~\mbox{GeV}$ and no *m*_T_ requirement are used, as no gain in sensitivity is obtained with a *m*_H_-dependent selection.

**Fig. 8:**
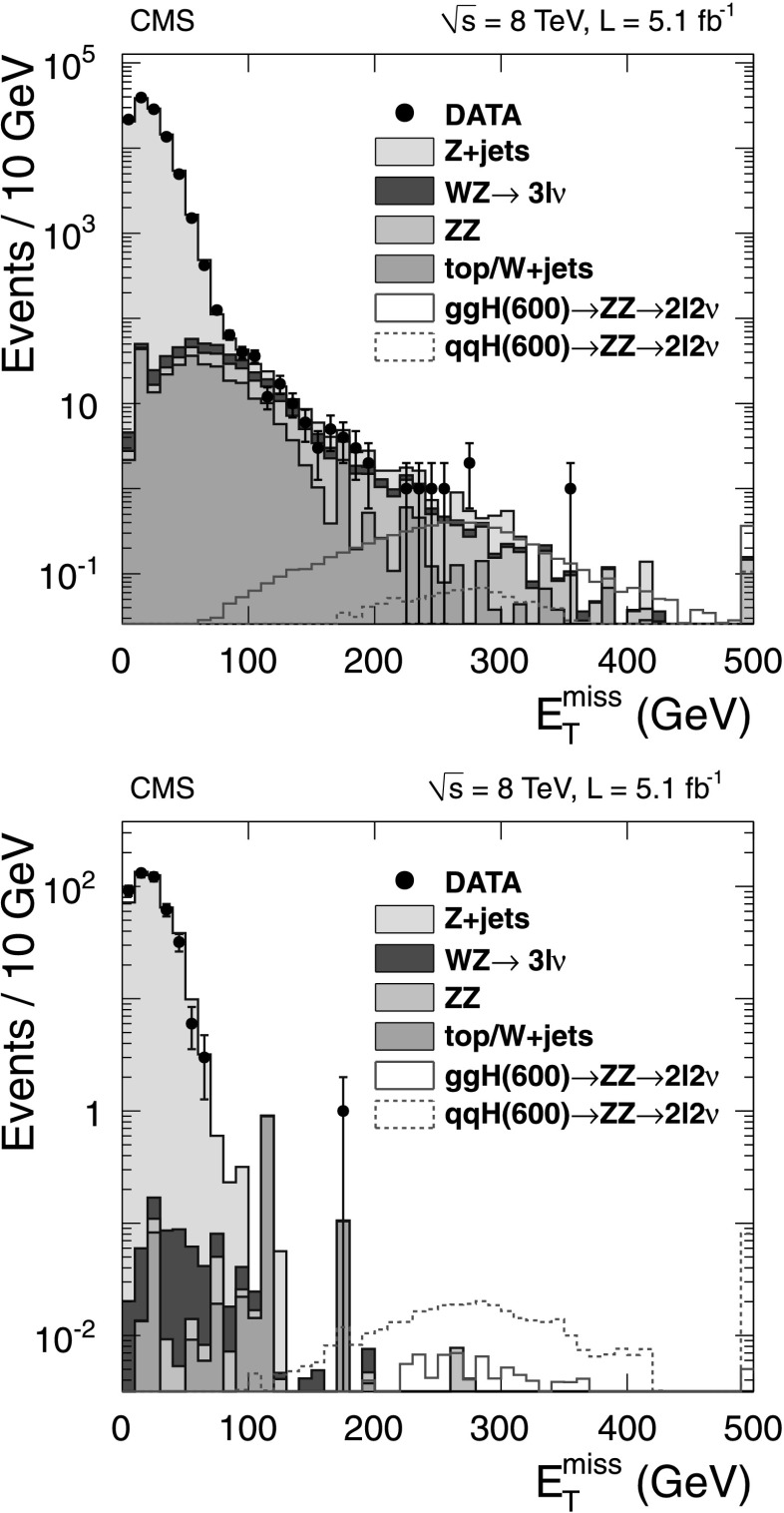
The $E_{\mathrm{T}}^{\text{miss}}$ distribution in data compared to the estimated background in the (*top*) gluon fusion and (*bottom*) VBF categories of the H→ZZ→2*ℓ*2*ν* channel. The dielectron and dimuon channels are combined. Contributions from ZZ, WZ, non-resonant background and Z+jets background are stacked on top of each other. The $E_{\mathrm{T}}^{\text {miss}}$distribution in signal events for *m*_H_=600 GeVis also shown. The last bin in each plot contain the overflow entries

The background composition is expected to vary with the hypothesised value of *m*_H_. At low *m*_H_, Z+jets and $\mathrm{t}\overline{\mathrm{t}} $ are the largest contributions, whilst at higher *m*_H_ (above 400 GeV), the irreducible ZZ and WZ backgrounds dominate. The ZZ and WZ backgrounds are taken from simulation [37, 61] and are normalized to their respective NLO cross sections. The Z+jets background is modeled from a control sample of *γ*+jets events. This procedure yields an accurate model of the $E_{\mathrm {T}}^{\text{miss}}$distribution in Z+jets events, shown in Fig. 8.

The uncertainty associated with the Z+jets background estimate is affected by any residual contamination in the *γ*+jets control sample from processes involving a photon and genuine $E_{\mathrm{T}}^{\text{miss}} $. This contamination could be as large as 50 % of the total Z+jets background. It is not subtracted, but assigned a 100 % uncertainty.

**Fig. 9:**
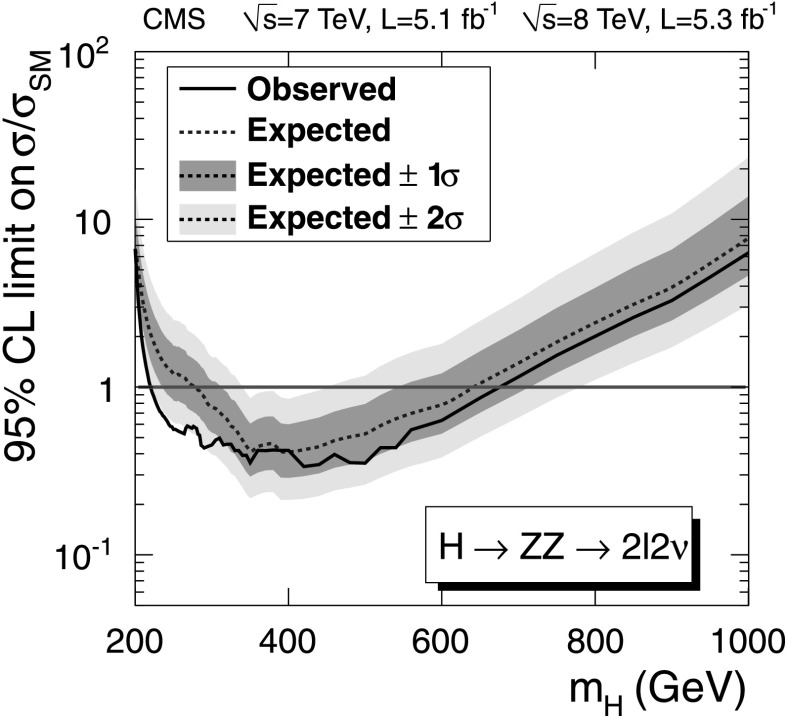
Observed (*solid line*) and expected (*dashed line*) 95 % CL upper limit on the ratio of the product of the production cross section and branching fraction to the SM expectation for the Higgs boson in the H→ZZ→2*ℓ*2*ν* channel

Background processes that do not involve a Z resonance (non-resonant background) are estimated with a control sample of events with dileptons of different flavor (e^±^ *μ* ^∓^) that pass the full analysis selection. This method cannot distinguish between the non-resonant background and a possible contribution from H→WW→2*ℓ*2*ν* events, which are treated as part of the non-resonant background estimate. This treatment considers only the H→ZZ channel as signal and is combined with the H→WW channel for the limit calculation. The interference between ZZ and WW channels is also taken into account [106]. The non-resonant background in the e^+^e^−^ and *μ* ^+^ *μ* ^−^ final states is estimated by applying a scale factor to the selected e^±^ *μ* ^∓^ events, estimated from the sidebands of the Z peak events (40<*m*_*ℓℓ*_<70 GeV and 110<*m*_*ℓℓ*_<200 GeV). The uncertainty associated with the estimate of the non-resonant background is evaluated to be 25 %. No significant excess of events is observed over the SM background expectation. The observed and expected upper limits as a function of *m*_H_ are shown in Fig. 9.

## Combined results

**Fig. 10:**
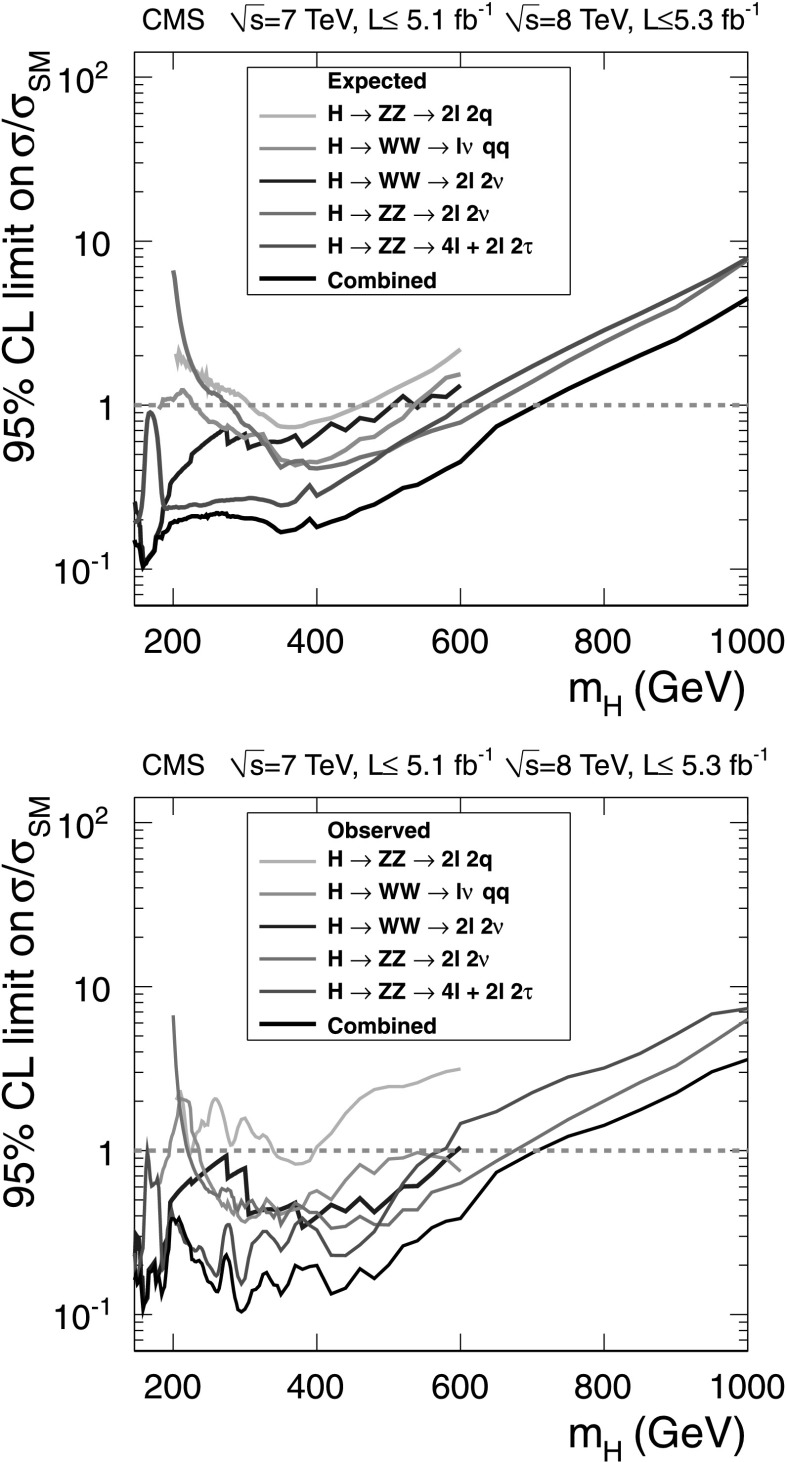
(*Top*) Expected and (*bottom*) observed 95 % CL limits for all individual channels and their combination. The *horizontal dashed line* at unity indicates the SM expectation

**Fig. 11:**
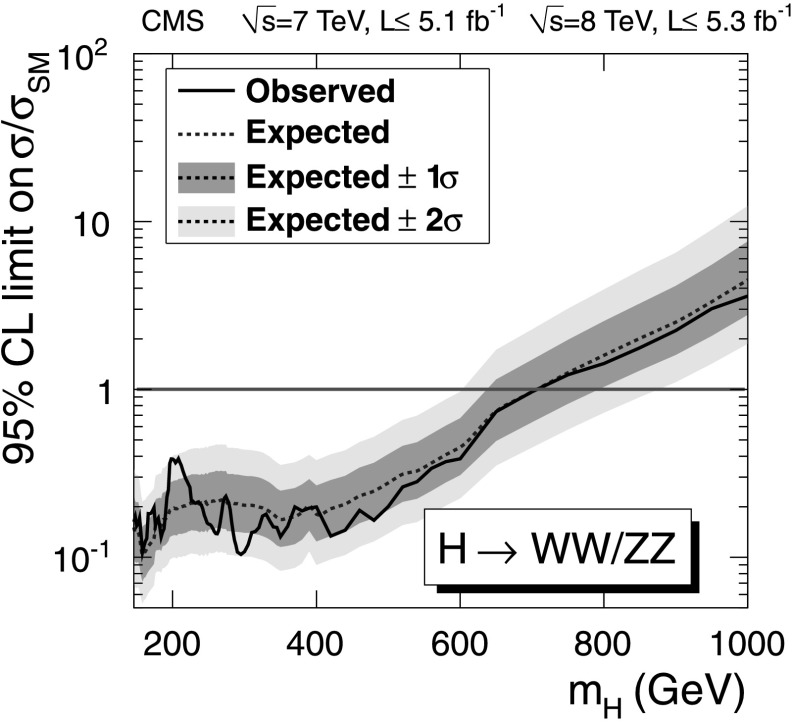
Observed (*solid line*) and expected (*dashed line*) 95 % CL upper limit on the ratio of the production cross section to the SM expectation for the Higgs boson with all WW and ZZ channels combined

The expected and observed upper limits on the ratio of the production cross section for the Higgs boson to the SM expectation, for each of the individual channels presented in this paper, are shown in Fig. 10. This figure also shows a combined limit, calculated using the methods outlined in Refs. [13, 82]. The combination procedure assumes the relative branching fractions to be those predicted by the SM, and takes into account the statistical and experimental systematic uncertainties as well as theoretical uncertainties. In the mass region 145<*m*_H_<200 GeV the branching fraction of the most sensitive channel, H→ZZ, is decreasing and has a typical dependence on *m*_H_, which is reflected in both the expected and observed limits. In this mass region the result of the combination is determined by the WW→*ℓνℓν* channel. At masses above 200 GeV the ZZ→2*ℓ*2*ℓ*′ channel becomes dominant, since low background contributions in this channel allow to keep high efficiency of the selection requirements. Starting at approximately 400 GeV the ZZ→2*ℓ*2*ν* starts to contribute significantly. The branching fraction of ZZ→2*ℓ*2*ν* is higher than ZZ→2*ℓ*2*ℓ*′, and the major background contributions decrease with *m*_H_ increase, thus allowing for selection requirements to be more and more effective in the 2*ℓ*2*ν* channel. The combined observed and expected limits agree well within uncertainties as shown in Fig. 11.

The previously expected exclusion range at 95 % CL, 118–543 GeV, is extended up to 700 GeV. Previously published results exclude at 95 % CL the SM-like Higgs boson in the range 127<*m*_H_<600 GeV [13]. The results of this analysis extend the upper exclusion limit to *m*_H_=710 GeV.

## Summary

Results are presented from searches for a standard-model-like Higgs boson in H→WW and H→ZZ decay channels, for Higgs boson mass hypotheses in the range 145<*m*_H_<1000 GeV. The analysis uses proton-proton collision data recorded by the CMS detector at the LHC, corresponding to integrated luminosities of up to 5.1 fb^−1^ at $\sqrt{s} = 7~\text{TeV}$ and up to 5.3 fb^−1^ at $\sqrt{s} = 8~\text{TeV}$. The final states analysed include two leptons and two neutrinos, H→WW→*ℓνℓν* and H→ZZ→2*ℓ*2*ν*, a lepton, a neutrino, and two jets, H→WW→*ℓν*qq, two leptons and two jets, H→ZZ→2*ℓ*2q, and four leptons, H→ZZ→2*ℓ*2*ℓ*′, where *ℓ*=e or *μ* and *ℓ*′=e or *μ*, or *τ*. The results are consistent with standard model background expectations. The combined upper limits at 95 % confidence level on products of the cross section and branching fractions exclude a standard-model-like Higgs boson in the range 145<*m*_H_<710 GeV, thus extending the mass region excluded by CMS from 127–600 GeV up to 710 GeV.
